# Ultradeformable Vesicles for Wound Healing: Ethosomes, Transferosomes, and Transethosomes in Topical Drug Delivery

**DOI:** 10.3390/pharmaceutics18030361

**Published:** 2026-03-13

**Authors:** Shery Jacob, Namitha Raichel Varkey, Anroop B. Nair

**Affiliations:** 1 Department of Pharmaceutical Sciences, College of Pharmacy, Gulf Medical University, Ajman 4184, United Arab Emirates; 2025mdd01@mygmu.ac.ae; 2 Department of Pharmaceutical Sciences, College of Clinical Pharmacy, King Faisal University, Al-Ahsa 31982, Saudi Arabia; anair@kfu.edu.sa

**Keywords:** wound healing, topical, vesicles, ethosomes, transferosomes, transethosomes, compositions, characterization

## Abstract

Wound healing is a dynamic and multifaceted biological process involving hemostasis, inflammation, proliferation, and tissue remodeling. Topical therapy is widely preferred for wound management due to its localized action and reduced systemic adverse effects. However, the effective delivery of therapeutic agents is often limited by the skin’s barrier properties, the complex wound microenvironment, and the physicochemical characteristics of drugs. This review highlights the key physicochemical parameters governing topical drug delivery in wound therapy, including drug solubility, molecular size, lipophilicity, vesicle size distribution, surface charge, encapsulation efficiency, lipid composition, ethanol concentration, and vesicle deformability, which collectively influence drug permeation and retention at the wound site. Nanovesicular delivery systems have emerged as promising strategies to overcome these limitations. In particular, ultradeformable vesicles such as ethosomes, transferosomes, and transethosomes have demonstrated enhanced skin permeation and improved drug deposition in periwound tissue due to their flexible membrane structure and optimized physicochemical properties. This review systematically discusses the composition, preparation techniques, and critical formulation parameters of these vesicular systems that determine their stability, elasticity, and permeation performance. Furthermore, their applications in delivering anti-inflammatory drugs, antimicrobial agents, bioactive phytochemicals, and regenerative therapeutics for different wound types are examined. Widely used in vitro, ex vivo, and in vivo evaluation methods, including permeation studies and wound healing models such as excision, burn, infected, and diabetic wounds, are also summarized. Finally, the review outlines current challenges related to formulation standardization, physicochemical characterization, safety assessment, and large-scale production, while highlighting the future potential of ultradeformable vesicles as next-generation nanocarriers for advanced wound healing therapies.

## 1. Introduction

The skin is one of the largest organs of human beings, with a typical surface area of around 2 m^2^ and contributing about 15–20% of the total body weight. It functions as a fundamental protective barrier between the internal environment and exterior physical, chemical and biological threats [1]. The skin has three main layers, namely, the epidermis, dermis and hypodermis. The epidermis is primarily composed of keratinocytes, which serve as a physical and immunological barrier. The dermis consists of connective tissue, blood vessels, nerves, fibroblasts and extracellular matrix (ECM) components necessary for mechanical strength and elasticity [2]. The hypodermis, which is primarily composed of adipose tissue, aids in insulation, cushioning and metabolic regulation. Apart from its barrier role, the skin is required for thermoregulation, sensory perception, immune defense and wound healing; therefore, its health is dependent on its integrity. Endogenous pathological conditions such as metabolic irregularities, vascular insufficiency, inflammation and infection, along with exogenous mechanical, thermal, chemical and surgical injuries, can have an impact on cutaneous tissue structure and function [3]. These disruptions can result in the development of skin wounds that can damage the skin barrier, increase the chance of microbial invasion, impair tissue regeneration, and eventually cause morbidity and even deadly risks if left untreated.

The recent epidemiological data indicate that dermatological disorders are among the most frequent types of human disease affecting approximately one third of the world’s population. The World Health Organization and Global Burden of Disease have reported that skin and subcutaneous diseases continue to be a major global public health concern, accounting for millions of disability-adjusted life years and billions of incident cases annually [4,5]. The worldwide impact of managing both acute and chronic wounds is constantly increasing. This increase is due to rising life expectancy, population aging, nutritional/immunological inadequacies and the growing number of comorbidities such as diabetes mellitus, obesity, vascular diseases and immobility. Such systemic disorders can disturb essential cellular and molecular processes, such as hemostasis, inflammation, angiogenesis and tissue remodeling, which generally results in delayed or nonhealing wounds [6]. Moreover, the increase in the prevalence of multidrug-resistant microorganisms worsens the situation by increasing the incidence, severity and duration of both acute and chronic wounds. In addition, high bacterial bioburden and biofilm formation are common characteristics of chronic wounds, which decrease the efficiency of traditional antimicrobial therapy and slow tissue regeneration. According to epidemiological research, approximately one in every four patients with diabetes mellitus will develop diabetic foot ulcers at some point during their life. Many of these patients acquire wound infections, which frequently result in disastrous implications such as osteomyelitis, limb ischemia and lower-extremity amputation [7]. Furthermore, skin ulcers linked to systemic sclerosis (scleroderma) are a unique and difficult type of chronic lesion. Microvascular dysfunction, tissue hypoxia, fibrosis and compromised immunological responses are the key causes of these lesions’ poor healing outcomes [8]. In such instances, localized therapeutic approaches, including wound dressings and tailored antibacterial or regenerative treatments, are necessary to promote healing, prevent infection and improve patients’ quality of life. Wound care is linked to longer treatment times, more frequent hospitalizations, higher risk of infection and recurrence, as well as high healthcare expenditures. This in turn results in a considerable socioeconomic burden on patients and healthcare systems worldwide. Outcomes differ significantly across different patient populations and wound types. The current clinical interventions frequently exhibit limited and inconsistent efficacy across diverse patient populations and wound types, despite significant advancements in the understanding of cutaneous repair mechanisms and the establishment of different types of treatment. This underscores the unmet need for more effective and targeted wound healing strategies.

## 2. Molecular Mechanisms of Wound Healing Process

Skin wound healing is a complex and carefully regulated biological process that involves the simultaneous activation of cellular, humoral and molecular pathways [9]. Inflammatory regulation, angiogenesis, re-epithelialization, collagen deposition and ECM remodeling are governed by growth factors, cytokines and signaling pathways. Dysregulation of these processes can impede healing, emphasizing the need for innovative therapeutic techniques to improve and accelerate tissue repair, such as personalized drug delivery systems and nanotechnology-based formulations [10]. Intrinsic injuries can appear as open wounds, which are characterized by disruption of the skin surface, or closed wounds, which injure underlying tissues or organs without breach of the skin surface. Wounds are closed using either regenerative processes that restore the original tissue architecture or reparative mechanisms that result in structural and functional recovery. The hallmark of regenerative healing is the restoration of normal tissue structure and function, while skin healing usually happens through fibrotic repair, indicated by collagen deposition, ECM remodeling and eventually scar formation [11].

## 3. Stages of Wound Healing Process

**Figure 1:**
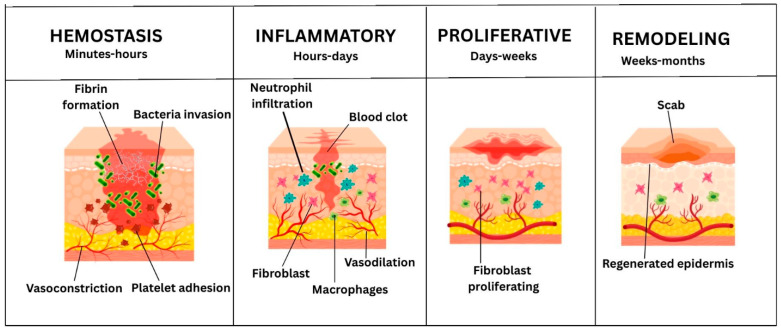
Different phases of wound healing.

The dynamic and organized biological process of wound healing consists of four overlapping phases: hemostasis, inflammation, proliferation and remodeling or maturation (Figure 1).

### 3.1. Hemostasis

This stage is the immediate response to vascular injury and involves rapid vasoconstriction of damaged blood vessels to reduce blood loss, mediated by endothelin, catecholamines, prostaglandins, and platelet-derived growth factor (PDGF) released from activated platelets [12,13]. When endothelial damage exposes the thrombogenic subendothelial matrix, platelets adhere, activate, and aggregate through integrin-mediated signaling, leading to cytoskeletal changes and formation of the primary platelet plug [14,15]. Platelet activation releases mediators such as ADP, thromboxane A_2_, cytokines, and growth factors that promote platelet recruitment and signaling for wound repair. Platelets also support the coagulation cascade, leading to thrombin formation and the conversion of fibrinogen into cross-linked fibrin, which stabilizes the platelet plug [16,17]. The fibrin-rich thrombus creates temporary ECM with fibronectin, vitronectin and thrombospondin. This promotes inflammatory cell recruitment, angiogenesis and stromal cell proliferation through mediators like transforming growth factor β (TGF-β), vascular endothelial growth factor (VEGF), PDGF, C-C Motif Chemokine Ligand 5 (CCL5) and complement activation, allowing progression to the next stages of wound healing [18].

### 3.2. Inflammatory Phase

The inflammatory phase begins after hemostasis and involves the coordinated recruitment of immune cells, including neutrophils, macrophages, lymphocytes, and mast cells. It is characterized by vasodilation, increased vascular permeability, and mediator release that promote leukocyte recruitment and immune activation [19]. Neutrophils arrive first to provide antimicrobial defense (reactive oxygen species (ROS), proteases, and antimicrobial peptides (AMPs)) and then undergo caspase- and cathepsin D-dependent apoptosis to support inflammation resolution [20]. Monocytes are recruited (e.g., via CC chemokine ligand 2 (CCL2)) and differentiate into macrophages, which clear apoptotic cells, present antigens and secrete cytokines/growth factors (TGF-α, TGF-β, PDGF, bFGF, and VEGF) that drive fibroblast proliferation, ECM deposition and angiogenesis [21,22]. Although macrophages play an important role in wound closure, excessive or prolonged inflammation can impair healing. Some studies show that wounds can be repaired with minimal inflammation and reduced scarring, suggesting that inflammation influences healing outcomes [23]. In diabetes, dysregulated inflammation and impaired macrophage polarization promote chronic inflammation, delayed closure and pathological scarring [24]. Resolution is actively regulated by pro-resolving lipid mediators (resolvins, protectins, lipoxins, and maresins), and B/T lymphocytes plus mast cells further shape immune responses, angiogenesis and fibrosis [25]. Acute wound secretions tend to support fibroblast DNA synthesis and proliferation, whereas chronic wound fluids can suppress fibroblast growth. These wound fluid mediators affect fibroblasts [26]. Chronic wounds often show persistent inflammation with elevated interleukin-1beta (IL-1β)/tumor necrosis factor-alpha (TNF-α) and heightened protease activity, degrading growth factors/ECM and delaying transition to proliferation [27]. Therapeutic strategies currently studied include targeting cytokine pathways (IL-1/TNF), inflammasome-related approaches, chemokine-axis modulation (MCP-1/CCR2) and iron-handling/chelation to reduce inflammation and restore repair [28]. Emerging approaches for wound management include ECM-based dressings, controlled-release therapies, and advanced profiling methods (proteomic, microbiome, and single-cell methods) to study immune dysregulation in chronic wounds [29].

### 3.3. Proliferative Phase and Neovascularization

This phase follows inflammation and involves granulation tissue formation, angiogenesis, re-epithelialization, and immune regulation. Granulation tissue mainly consists of activated fibroblasts that produce extracellular matrix (ECM), supporting tissue repair and wound contraction [30]. In adults, angiogenesis is stimulated by hypoxia and growth factors such as VEGF and PDGF. This process involves endothelial cell activation, migration, proliferation, and capillary formation, with pericytes stabilizing new vessels to supply oxygen and nutrients for tissue repair [31]. This endothelial-driven process is regulated by proteases, growth factors and pathways involving VEGF, FGF, PDGF-B, TGF and angiopoietins [32]. TGF-β signaling coordinates inflammatory and stromal responses and influences cell proliferation/migration, angiogenesis and ECM deposition. Angiogenesis supports granulation tissue development and ECM remodeling [33]. Emerging strategies to improve angiogenesis in chronic wounds include localized nucleic acid (DNA/mRNA) delivery through dressings or scaffolds for sustained gene expression. Oxygen-releasing systems combined with ROS-scavenging hydrogels have also shown improved angiogenic signaling and accelerated wound closure in diabetic models [34]. Immune mediators can also enhance neovascularization, and C1q has been reported to stimulate angiogenesis through complement-independent mechanisms and to increase vessel density in wound healing models [35].

### 3.4. The Remodeling Phase

The remodeling phase is the longest stage of wound healing and determines scar formation and tissue strength. During this phase, granulation tissue is remodeled into mature scar tissue through ECM reorganization and replacement of collagen III with collagen I, increasing tensile strength [36]. Myofibroblasts regulate ECM turnover via matrix metalloproteinases, and their imbalance can contribute to chronic wounds or abnormal scarring [37]. Macrophages help resolve healing by removing excess ECM and debris, while newly formed blood vessels are reduced through endothelial apoptosis and regulatory mechanisms to establish stable vasculature [38]. Collagen is the main dermal ECM structural protein and is essential for restoring tensile strength. Fibroblasts/myofibroblasts synthesize collagen (especially types III and I) under pro-repair signaling such as TGF-β, with net accumulation governed by the balance of synthesis versus proteolysis [39]. In chronic wounds, increased protease activity can cause excessive extracellular matrix degradation and impair granulation tissue formation. Therefore, improving healing may involve reducing inflammation and proteolysis, using collagen or ECM-mimicking dressings, and enhancing the local delivery of repair-promoting signals [40]. Nutritional support also contributes to wound repair. For instance, vitamin C aids collagen hydroxylation and stability [41]. Collagen-based and nanoparticle-enhanced hydrogels are being developed to improve growth factor stability, controlled release, and support for fibroblast activity, angiogenesis, and collagen deposition. These systems help overcome the rapid degradation of soluble growth factors in wound environments.

## 4. Topical Drug Delivery

Topical drug delivery involves applying a formulation to the skin to produce therapeutic effects mainly at the application site. It is commonly used in dermatological and wound-related conditions because it enhances drug availability in superficial tissues while limiting systemic absorption and associated adverse effects [42]. In wound management, topical therapy is intended to maintain effective drug levels at the wound bed and in the periwound region to promote repair. Such formulations may influence the sequential phases of healing hemostasis, inflammation, proliferation and remodeling. They act by controlling infection and oxidative stress, dampening excessive inflammation and supporting granulation tissue formation, angiogenesis, re-epithelialization and collagen deposition. Therapeutic agents, including growth factors and cytokines, antibacterial agents, proteins/peptides, small molecules, phytochemicals and other bioactive compounds, can accelerate wound repair by enhancing the underlying physiological processes that drive healing [43]. Moreover, well-designed topical vehicles such as hydrogels can aid healing by sustaining a moist environment, increasing residence time and providing controlled release aligned with the dynamic requirements of the healing process [44,45].

### 4.1. Physicochemical Determinants of Passive Skin Permeation

Transport across the stratum corneum (SC) is feasible only for drugs with a favorable physicochemical profile. Passive transdermal candidates should have a molecular weight < 500 Da and moderate lipophilicity (log P ~1–3). They should also exhibit a substantial unionized fraction at skin/formulation pH (pKa-dependent), a relatively low melting point (<200 °C), and balanced solubility in both aqueous and lipid domains to enable release from the formulation and partitioning into SC lipids [46]. A drug should also be highly potent with a low daily dose (<10–20 mg/day) and must be stable and compatible with formulation components [47]. Studies indicate that drugs with higher polar surface areas exhibit increased hydrogen bonding interactions with skin components such as keratin and collagen, which consequently slow drug permeation through the skin. For example, insulin, with a high topological polar surface area of 159 Å^2^, demonstrates poor skin permeation, whereas minoxidil, having a much lower value of 42 Å^2^, exhibits good skin penetration [48]. Amorphous drugs enhance cutaneous transportation by assisting dissolution and permeation, whereas crystalline drugs have challenges in permeation due to their highly ordered molecular structure [49]. It is important to remember that a drug’s percutaneous absorption is determined by the combined and synergistic effects of numerous physicochemical features rather than by a single factor. Identifying the inconsistent distribution of drugs among various skin layers and entry into systemic circulation is critical for the proper characterization of cutaneous pharmacokinetics [50]. Furthermore, a detailed understanding of cutaneous metabolism and drug excretion through the skin is critical for optimizing topical and transdermal drug delivery systems [51].

### 4.2. Percutaneous Dermal Absorption

The SC is an ~5–20 µm thick outer skin layer that acts as the primary barrier to the external environment. The SC is a highly organized structure, which comprises around 10–15 layers of corneocytes embedded in continuous lipid matrix and is reinforced by corneodesmosomes and tight connections [52]. Variations in skin thickness and integrity over distinct body regions considerably influence absorption rates, due to changes in SC thickness, lipid content, moisture level and follicular density. Skin condition plays a significant role, with diseased, injured, or weakened skin demonstrating markedly increased permeability, notably for water-soluble and low-molecular weight chemicals compared to intact skin [53]. The initial step in percutaneous absorption is the release of a drug from the formulation vehicle into the SC, which is determined by the drug’s thermodynamic activity. Further, the capacity of a drug to permeate the SC is determined by its diffusivity and solubility relative to the formulation. The released drug will then partition into the SC before diffusing into the viable epidermis and dermis. The SC can function as a drug depot, retaining the applied drug and enabling its gradual release into the deeper skin layers and systemic circulation long after application [54]. Drug flux increases with higher values of the partition coefficient and saturated drug concentration in the vehicle. In addition, protein and lipid domains regulate solute uptake according to the drug’s lipophilicity [55]. Consequently, the main challenge in topical drug delivery is to modulate the SC barrier to enable drug penetration into the viable epidermis/dermis and achieve the desired therapeutic concentration within skin tissues while minimizing systemic absorption. Many prospective strategies have been investigated to overcome the SC barrier, including chemical methods (solvents/co-solvents, fatty acids, terpenes, essential oils, surfactants, sulfoxides, lactams, urea, ionic liquids, etc.) [56,57,58]. In addition, different physical methods, including microneedles, iontophoresis, electroporation, ultrasound/sonophoresis, laser or thermal ablation, photomechanical/pressure waves, needle-free jet/ballistic injection systems, etc., have been explored [59].

## 5. Topical Drugs and Strategies for Wound Healing

Several factors influence the selection of a topical administration for wound therapeutics. Impaired and heterogeneous wound bed vasculature can limit local perfusion and compromise drug exposure at the target site, making direct application to the wound bed advantageous [60]. Topical delivery also restricts systemic absorption, thereby reducing the adverse effects. In addition, the wound microenvironment is often rich in proteases and pro-inflammatory mediators, which can destabilize or inactivate susceptible agents, highlighting the need for protective formulations that maintain adequate local concentrations [61]. An efficient topical system should also ensure that drugs remain at the wound interface throughout the healing process.

Controlled drug delivery is the fundamental design principle for chronic wound therapy because nonhealing wounds are characterized by a microenvironment that is temporally and biochemically dysregulated. Impaired angiogenesis and prolonged hypoxia are common in chronic wounds, and these factors lead to poor transition from inflammation to proliferation and remodeling [62]. This persistent inflammatory state is caused by an imbalance of mediators as well as continuing immune cell recruitment and activation, which prolongs rather than heals tissue damage [63]. A key component of this pathology is the excessive protease burden, especially increased matrix metalloproteinases, frequently paired with inadequate regulation by endogenous inhibitors. This can promote degradation of provisional ECM and growth factors, thus compromising granulation tissue formation and re-epithelialization [64].

Given the phase-specific biology of healing, spatiotemporally programmed delivery has been proposed to redirect the chronic wound trajectory. This involves first releasing antimicrobials and/or anti-inflammatory signals to lower bioburden and dampen excessive inflammation, followed by presentation of pro-angiogenic and pro-regenerative signals (e.g., VEGF or FGF family growth factors, EGF, and supportive small molecules) to promote neovascularization, granulation tissue maturation and re-epithelialization [65]. Such a logic also aligns with the recognition that chronic wounds are often driven by intertwined cycles of hypoxia and inflammatory injury, including ischemia–reperfusion-associated damage, which further amplifies inflammatory signaling [66].

### 5.1. Anti-Inflammatory Agents

Chronic wounds such as diabetic foot ulcers are often persistently maintained in an inflammatory state; hence, anti-inflammatory therapy aims to reduce excessive cytokine/protease signaling and restore progression to repair. Current approaches include cytokine- and pathway-targeted strategies like inhibiting nuclear factor kappa B (NF-κB) and NOD-like receptor family pyrin domain containing 3 (NLRP3) inflammasome activity to reduce IL-1β-driven inflammation. These targets are described as promising in recent diabetic wound reviews and preclinical studies using small-molecule inflammasome inhibitors like MCC950 to improve closure [67]. In parallel, specialized pro-resolving mediators, namely, lipoxins, resolvins, protectins, maresins, cysteinyl and n-3 docosapentaenoic acid, actively promote resolution rather than simple immunosuppression through the process known as efferocytosis [68]. Poly(lactic-co-glycolic acid) microparticles encapsulating lipoxin A4 (LxA4) were developed for the treatment of skin ulcers. In a rat skin wound model, the formulation reduced inflammatory cell infiltration, accelerated wound closure, decreased IL-1β and TNF-α levels, and increased TGF-β levels [69]. A catechol chitosan hydrogel incorporating acetalized cyclodextrin nanoparticles loaded with resolvin D1 (RvD1) was developed for chronic wound treatment. In vitro testing using a macrophage cell line showed increased production of the anti-inflammatory cytokine IL-10, and in a rat wound model the hydrogel accelerated wound closure [70]. Topical corticosteroids are used mainly for inflammatory periwound dermatitis and occasionally to reduce excessive inflammation in highly inflamed wounds under specialist supervision with monitoring due to infection risk. Topical NSAIDs may reduce prostaglandin-driven inflammation, but their routine benefit in speeding chronic ulcer closure is limited [71].

### 5.2. Antimicrobial Agents

Topical antimicrobials are used in burn and wound care to prevent or control infection by reducing local microbial burden. However, their routine use is debated because colonization is common and does not necessarily prevent healing in progressing wounds [72]. Clinical guidance recommends selecting agents/dressings with an appropriate antimicrobial spectrum and acceptable local tolerability and matching product choice to wound depth, exudate level, and risk or signs of local infection. In the context of antimicrobial resistance and stewardship, topical antiseptic dressings, including silver-based products that release antimicrobial silver ions, are often considered for critically colonized or locally infected wounds. However, their efficacy and cytotoxicity and the emergence of resistance remain important considerations. Silver sulfadiazine is still widely used in many burn settings, but recommendations vary across guidelines and clinical scenarios. Examples of topical agents used in chronic wounds include cadexomer iodine, polyhexamethylene biguanide and silver-based antimicrobials (silver dressings), which are mainly used to reduce bioburden and support infection/biofilm management when indicated [73]. Examples of multifunctional dressings/platforms include collagen dressings and oxygenated regenerated cellulose dressings/collagen dressings, which provide an ECM-like scaffold and help to modulate the chronic wound microenvironment, including excess protease activity. In addition, advanced antimicrobial/antibiofilm dressings are designed to combine microbial control with structural and biological support of the wound bed [74].

### 5.3. Antimicrobial Peptides (AMPs)

AMPs are small, post-translationally processed peptides that are fundamental components of the innate immune response, rapidly mobilized in response to pathogenic and environmental signals to provide early host defense. These cationic peptides exhibit broad-spectrum antimicrobial activity, directly targeting bacterial membranes and intracellular processes. They also modulate immune responses through chemotaxis and regulation of chemokines and receptors, thereby influencing inflammation and wound healing pathways [75]. Recently, *RWPIL (Arg-Trp-Pro-Ile-Leu)*, a short cationic AMP was identified using bacterial membrane chromatography and subsequently formulated into an oxidized dextran-based hydrogel. It demonstrated potent antibacterial activity against both *Escherichia coli* and *Staphylococcus aureus* and significantly enhanced epithelial adhesion and wound healing in an infected wound model [76]. Despite this promise, clinical translation has been constrained by challenges, including proteolytic instability, limited in vivo bioavailability and cytotoxicity, which complicate therapeutic use [77]. Furthermore, the discovery of transferable resistance mechanisms like plasmid-mediated mcr genes (mobile colistin resistance genes mcr-1 to mcr-10) has raised doubts about the widely held belief that AMPs are unlikely to cause resistance. This evidence emphasizes the potential for resistance to develop against AMP-based treatments as well [78]. To deal with these barriers, modern approaches involving chemical modification, structural optimization and new delivery systems are being studied. Additionally, topical AMP candidates have produced inconsistent clinical results, with LL-37 showing improved healing in venous leg ulcers at optimal dosing. However, pexiganan failed to meet phase III endpoints in trials that involved diabetic foot ulcers, illustrating the vital importance of formulation and dosage in AMP therapeutic development [79]. Advances in bioengineering and peptide design continue to support the development of AMP drug candidates, indicating their promise as substitutes or adjuncts to traditional antibiotics in the treatment of resistant infections and nonhealing wounds.

### 5.4. MicroRNAs (miRNAs)

miRNAs are essential post-transcriptional regulators that regulate gene expression in wound healing by either supporting or inhibiting particular wound repair pathways. Their expression patterns vary significantly between acute healing wounds and chronic nonhealing wounds, and they shift dynamically during the healing phases [80]. For instance, miR-92a is overexpressed in nonhealing wounds, which reduces angiogenesis, whereas suppression of miR-92a has been demonstrated to promote angiogenesis and expedite healing in animal models. Wound healing has been linked to a number of other miRNAs. A prospective therapeutic target, miR-21, is upregulated after skin damage and regulates fibroblast proliferation, angiogenesis, collagen production, anti-inflammatory responses and re-epithelialization [81]. miR-146a is important in the inflammatory phase of healing because dysregulation of miR-146a is linked to prolonged inflammation in diabetic ulcers, and its reduction has been investigated for improving wound closure. miR-155 regulates immune responses during wound inflammation, and decreasing it leads to a reduction in inflammatory cell accumulation and improvement in healing [82]. While miR-29 regulates collagen deposition and ECM remodeling, which are required to prevent excessive scarring, other miRNAs, such as miR-126, stimulate angiogenesis. All of these findings indicate the possibility of employing mimics or inhibitors to target specific miRNAs in order to promote regeneration and fix dysregulated gene expression in chronic wounds [80].

### 5.5. Growth Factors

Recent developments in wound healing have led to the discovery of several growth factors that regulate key events across the inflammatory, proliferative and remodeling phases [83]. Nevertheless, PDGF is still the only growth factor that the FDA has approved for the treatment of chronic nonhealing wounds. Recombinant PDGF-BB (becaplermin) has exhibited improved healing outcomes in diabetic foot ulcers in clinical trials and retrospective investigations. However, its normal clinical use is limited, mainly due to cost and practical reasons [12]. A major problem for growth factor-based therapies is the hostile proteolytic environment of persistent wounds, especially diabetic foot ulcers. These wounds show increased matrix metalloproteinase activity and decreased endogenous inhibitors, resulting in rapid degradation and limited bioavailability of externally applied proteins [84]. In order to solve these limitations, various delivery techniques such as intralesional injections, topical sprays, sustained-release dressings, hydrogels, gene therapy and platelet-rich plasma have been examined. However, these studies showed mixed results. Topical EGF sprays and intralesional release of factors such as GM-CSF have been shown to promote healing in chronic wounds, while other treatments like bFGF sprays have not fulfilled therapeutic goals [85]. Platelet-rich plasma and gene therapy aim to reestablish the wound microenvironment by promoting the local or autologous production of multiple regenerative mediators. On the other hand, sustained-release systems and protease-modulating dressings seek to extend growth factor activity. In general, these approaches demonstrate the therapeutic effectiveness of growth factor-based therapies in chronic wound healing as well as the ongoing translational challenges.

### 5.6. Chemokines

Preclinical studies indicate that chemokines play an important role in wound healing by increasing immune cell recruitment and tissue restoration [86]. Topical CCL2 treatment promoted diabetic mouse wound healing by restoring normal closure, collagen deposition and neovascularization. This effect was associated with enhanced infiltration of macrophages expressing VEGF and TGF-β, increased recruitment of endothelial progenitor cells, and acceleration of angiogenesis at the wound site [87]. Despite promising outcomes, the clinical translation of gene- and cell-based chemokine delivery systems remains constrained by regulatory and feasibility challenges. Research findings indicate mesenchymal stromal cell-derived CCL2 as a critical mediator of MSC-induced accelerated wound healing and highlight its potential as a therapeutic target to enhance MSC-based wound repair strategies [88]. A major recent shift is toward sustained, localized chemokine delivery platforms that avoid frequent dressing changes while preserving bioactivity. The most advanced example is a live biotherapeutic platform. The CXCL12-expressing *Limosilactobacillus reuteri* (ILP100-Topical) has now progressed beyond animal studies to a first-in-human clinical study demonstrating safety/tolerability with exploratory signals on wound repair [89]. A subsequent phase IIa diabetic foot ulcer trial was registered (NCT05608187), although it was terminated due to recruitment issues, highlighting real-world translational barriers despite a strong mechanistic rationale. Indeed, ILP100 has shown in vitro activity against highly multidrug-resistant wound pathogens, suggesting potential value in wounds complicated by resistant infections [90]. This dual function is an additional emerging benefit of this designed probiotic strategy.

### 5.7. Stem Cell-Based Therapy

Stem cell-based therapy is an expanding strategy in regenerative medicine because stem cells can self-renew and differentiate. This therapy also promotes repair through strong paracrine signaling (release of cytokines, growth factors and extracellular vesicles) [91]. In skin regeneration and chronic wound management, the most widely investigated cell sources include adult mesenchymal stromal/stem cells (MSCs), embryonic stem cells and induced pluripotent stem cells, with MSCs receiving particular attention due to their immunomodulatory and pro-angiogenic effects. For chronic wounds, stem cell therapies are proposed to accelerate repair by enhancing angiogenesis and re-epithelialization, modulating excessive inflammation and stimulating immune and stromal repair programs [92]. Increasingly, cell-free approaches such as MSC-derived exosomes are being explored because they can reproduce many regenerative benefits (e.g., effects on fibroblasts, keratinocytes, immune cells and endothelial cells) while potentially reducing practical barriers associated with live-cell delivery [93].

### 5.8. Phytochemicals

Many medicinal plants and plant-derived phytochemicals support wound repair because they combine antioxidant, anti-inflammatory, immunomodulatory and antimicrobial actions [94,95]. By scavenging ROS and limiting lipid peroxidation, these agents can reduce oxidative injury and create microenvironment that favors fibroblast proliferation, granulation tissue formation, angiogenesis and provisional ECM deposition. Major antioxidant classes include polyphenols (flavonoids, tannins, and phenolic acids), quinones (e.g., naphthoquinones/anthraquinones), terpenoids and saponins. Mechanistically, several botanicals appear to activate key repair mediators by upregulating pro-healing pathways (e.g., VEGF-linked angiogenesis and TGF-β-related matrix deposition) while downregulating excessive inflammatory signaling (e.g., TNF-α, IL-1β, and inducible nitric oxide synthase), helping shift wounds from persistent inflammation to proliferation/remodeling. For instance, *Centella asiatica*, which contains asiaticoside/madecassoside, has been reported to promote wound repair by enhancing TGF-β/Smad-mediated matrix deposition and VEGF-associated angiogenesis, while reducing pro-inflammatory cytokines such as TNF-α and other inflammatory mediators [96]. Curcumin is widely studied for cutaneous repair and may support healing by modulating inflammation and oxidative stress while promoting fibroblast activity, granulation tissue formation and collagen organization [97]. Many experimental findings also suggest that curcumin may improve scar quality by regulating fibroblast activation and ECM remodeling [98,99]. Honey has been found to modulate inflammatory cytokines, including TNF-α and IL-1β, while supporting processes linked to granulation and angiogenesis [100]. Aloe vera remains a commonly used topical botanical for burns and wounds [101]. Contemporary reviews attribute its effects to a mixture of bioactives, including anthraquinones, saponins, flavonoids and polysaccharides such as acemannan. They exhibit antioxidant and immunomodulatory activity and are being developed in biomaterial formats for wound applications [102]. Plant-based essential oils/terpenoids and their active compounds are increasingly being explored as adjuncts for chronic or infected wounds. This is because they combine antimicrobial, anti-inflammatory and antioxidant actions that can help control bioburden and reduce excessive inflammation. It is also emphasized that these volatile bioactives (e.g., monoterpenes/terpenoids such as thymol, carvacrol, eugenol, linalool, etc.) are being incorporated into modern delivery systems. These include nanoformulations and bioactive dressings (hydrogels, nanofibers, and films) to improve stability, controlled release, antibiofilm performance, and overall wound closure and tissue regeneration outcomes [103].

### 5.9. Adjunctive Wound Healing Therapies

Non-endogenous agents continue to play an important role in wound management, targeting infection control, inflammation, tissue regeneration and perfusion. Although silver dressings are widely used for antimicrobial control, high-quality evidence for improved healing or infection outcomes remains inconsistent [104]. Because ischemia and microvascular dysfunction are common in chronic wounds, topical vasodilator strategies have been explored. Isosorbide dinitrate spray combined with chitosan gel has been clinically studied in diabetic foot ulcers to enhance local perfusion and repair [105]. Nitric oxide is being delivered via hydrogels and advanced platforms to combine antimicrobial action, angiogenesis support and immunomodulation with localized sustained release [106]. Constant efforts by researchers have resulted in the development of smart dressings that can sense, distribute active drugs and adapt themselves. These dressings improve drug bioavailability on the wound surface by detecting inflammatory signals such as temperature, pH and oxygen content [107].

## 6. Nanovesicles

Nanosized vesicular delivery systems offer some of the most promising ways of accessing the SC and can increase the bioavailability of encapsulated actives and enable regulated therapeutic activity [108,109]. Various vesicular carriers, including niosomes, liposomes, transferosomes, ethosomes, transethosomes, cubosomes and invasomes, are widely used nanosystems [110]. These vesicles are capable of extending the drug residence time in the epidermis by producing local depots and favoring partitioning into the SC and viable epidermis [111]. Meanwhile, they also modify the systemic absorption by controlling drug release kinetics and diffusion across the epidermal barrier. This in turn reduces systemic exposure and enhances local targeting [112]. The performance of these carriers is generally influenced by vesicle size and deformability, surface charge, lipid/surfactant composition and drug carrier affinity, which together affect skin contact, follicular deposition and depth of penetration [112].

Apart from improving delivery, nanovesicles are able to assist in wound healing by maintaining local drug levels, enhancing moisture balance when incorporated into hydrogels or dressings. They also modify important healing pathways, such as in chronic and diabetic wound models. This in turn reduces excessive inflammation while promoting fibroblast activity, re-epithelialization, angiogenesis and organized collagen deposition [113]. Furthermore, exosomes and exosome-like nanovesicles are physiologically active vesicles that contain proteins and regulatory RNAs. They may modify wound cell responses by improving angiogenesis and re-epithelialization while also assisting in matrix remodeling. When coupled with dressings (such as hydrogels), these vesicles may improve local retention and therapeutic durability in chronic wounds [113,114]. Plant-based nanovesicles have gained popularity as wound healing therapies, especially for chronic diabetic wounds. Such nanovesicles are stable, easily accessible and low-immunogenic carriers of bioactive molecules that can help in wound healing, particularly in chronic diabetic wounds. They promote cell migration and proliferation, reducing inflammation and infection and increasing angiogenesis.

## 7. Ultradeformable Vesicles: Ethosomes, Transferosomes and Transethosomes

Ultradeformable vesicles like ethosomes, transferosomes and transethosomes offer numerous benefits over traditional vesicular carriers like liposomes and niosomes, especially for topical drug administration and wound healing. Their unique composition imparts exceptional membrane flexibility, enabling them to deform and permeate through the narrow intercellular pathways of the SC while remaining intact, which allows efficient delivery of therapeutic agents to deeper skin layers. They differ mainly in their composition, permeation mechanisms and ability to permeate deeper skin layers. Table 1 compares the different properties of ethosomes, transferosomes and transethosomes.

Ultradeformable vesicles differ mechanistically from commonly used biopolymer-based wound delivery systems (e.g., collagen, alginate, chitosan, and hyaluronic acid dressings). Typically, ultradeformable vesicles enhance skin permeation through bilayer flexibility and fluidization of stratum corneum lipids. In contrast, most approved biopolymer wound dressings maintain a moist environment, absorb exudate, and support tissue regeneration, with drug release mainly occurring through diffusion or matrix degradation rather than active penetration enhancement [115]. Biopolymers such as alginate, chitosan, and hyaluronic acid also contribute to wound repair through intrinsic biological properties, including gel formation, hemostatic activity, antimicrobial effects, and extracellular matrix support [116]. In summary, ultradeformable vesicles enhance drug delivery across skin barriers, whereas biopolymer-based systems mainly support wound protection and physiological healing at lower cost and with established clinical use [117,118].

### 7.1. Ethosomes

Ethosomes are newly developed, transformed, lipid-based vesicular carriers prepared using phospholipids, water and ethanol. Indeed, ethosomes are unique because they contain large amounts of ethanol (~20–45% *v*/*v*), which improves skin penetration by disturbing the SC lipids’ ordered structure and increasing the lipid bilayer fluidity and deformability [119]. Various properties of ethosomes are summarized in Table 1. Phospholipids and ethanol are required for the self-assembly and structural integrity of ethosomal vesicles. They produce amphiphilic bilayers that encapsulate drugs while maintaining vesicle architecture. The aqueous core improves the solubilization and stabilization of both hydrophilic and lipophilic drugs within the carrier system. This type of vesicle is frequently used in pharmaceutical formulations to enhance drug/phytochemical delivery for both localized and systemic therapeutic effects. When compared to conventional vesicular systems such as liposomes, ethosomes have a notably higher quantity of ethanol, which functions as a powerful penetration enhancer [120,121]. Ethosomes offer several advantages that make them suitable for cutaneous and transdermal drug delivery, including high drug entrapment efficiency, improved physical stability and low vesicle aggregation [122]. In addition to enhancing drug bioavailability by dual mechanisms (SC lipid disruption and increased SC fluidity), the high ethanol level results in remarkable vesicular deformability, allowing for deeper skin penetration. Ethosomal systems are highly efficient in the treatment of dermatological disorders like psoriasis, microbial infections, cutaneous melanoma and wound healing due to their controlled drug deposition in different layers of the skin [123]. Additionally, by promoting localized drug delivery, ethosomes reduce systemic side effects and can work well with various topical dosage forms, including gels and creams, in addition to transdermal drug delivery systems such as patches and microneedles. Furthermore, ethosomal systems can be developed with very simple, reproducible processes that are easily scalable from lab to industrial production, allowing for cost-effective, large-scale manufacturing while preserving formulation stability and performance [119]. They greatly improve patient compliance and have a wide range of pharmaceutical and cosmetic uses due to their noninvasiveness, acceptable safety profile and relatively simple commercialization. Ethanol reduces lipid packing density and increases membrane permeability through interacting and fluidizing SC intercellular lipid domains. This action increases drug transport across the SC and allows ethosomal vesicles to penetrate deeper intracellularly and intercellularly. It is noteworthy that the addition of ethanol to semisolid formulations, such as creams, substantially alters dermal permeability, which depends on the kind of skin. Additionally, ethanol reduces permeation and increases transepidermal water loss in intact skin, while exerting minimal impact on irritated skin [124]. These effects are triggered by ethanol-induced barrier changes, which include the development of a transient pudding skin layer that limits penetration. The findings emphasize the importance of carefully managing ethanol levels in ethosomes.

Ethosomal vesicles are soft and highly fluid, which can induce physical instability during storage, leading to aggregation, fusion and drug leakage. While the high concentration of ethanol is good for penetration, it may result in skin irritation or dryness, particularly with continuous use or in sensitive people [125]. In addition, ethosomes may have a comparatively short shelf life. However, stability can be maintained by carefully altering formulation factors. There are certain concerns about reproducibility, long-term storage stability and large-scale manufacturing [126].

#### 7.1.1. Types of Ethosomes

Based on their composition and functional changes, ethosomal systems are classified as classical ethosomes, binary ethosomes, transethosomes, composite phospholipid ethosomes and active targeting ethosomes [127]. Classical ethosomes are produced from phospholipids, ethanol and water. However, binary ethosomes include additional alcohol, such as propylene glycol or isopropyl alcohol, while transethosomes comprise edge activators or surfactants that enhance vesicle deformability and skin penetration [128]. Each category has distinct physical attributes, permeation behaviors and biological benefits that are meant to solve particular formulation and therapeutic challenges. These structural and compositional differences result in controlled skin penetration, improved vesicular stability and deformability, optimal drug loading, and tailored delivery to certain skin layers or disease areas [129].

Classical ethosomes are stable lipid-based vesicular carriers comprising phospholipids, cholesterol, ethanol and water in various ratios. From a formulation and mechanistic standpoint, the higher efficacy of ethosomes over traditional vesicular carriers such as liposomes may be due to basic variations in vesicle composition and interaction with the epidermal barrier [130]. Ethosomes are more efficient than rigid liposomes in crossing the SC’s small intercellular spaces owing to their high ethanol concentration, which additionally increases lipid bilayer fluidity and vesicle deformability [131]. Furthermore, ethanol damages the skin complex lipid domains, decreasing barrier resistance and enabling intact vesicles to travel deeper. As a result, ethosomes are better than liposomes with regard to drug loading efficiency, skin retention and transdermal flux, particularly for drugs that have low intrinsic skin permeability. These features enable ethosomes to be more efficient transdermal delivery systems for delivering active compounds. When ethosomal and liposomal vesicles loaded with psoralen were evaluated, the ethosomes exhibited significantly higher transdermal permeability (3.50 times higher) and dermal drug accumulation (2.15 times higher) than regular liposomal vesicles [132]. In a separate study, rosmarinic acid-loaded liposomes and ethosomes were developed and compared. The ethosomal formulations showed significantly higher drug permeation across human skin, along with greater transdermal flux, than liposomal systems [133].

Binary ethosomes are an advanced modification of conventional ethosomes in which a cosolvent, usually propylene glycol, is utilized to replace a certain amount of ethanol to generate an ethanol/propylene glycol mixture. By improving vesicular flexibility and fluidizing the SC lipid matrix, the two alcohols work in combination to improve drug retention and skin permeability as compared to conventional ethosomes. Binary ethosomes show better skin biocompatibility while maintaining efficient epidermal penetration, in contrast to transethosomes, which contain surfactants that may irritate the skin. The additional alcohol improves the stabilizing action of ethanol by enhancing vesicle fluidity, stability and aggregation tendency. This compositional modification increases drug solubility, vesicular stability and penetration efficiency, while lowering ethanol volatility and possible skin irritation [134]. Propylene glycol also improves vesicle flexibility and contact with the SC by serving as a humectant and permeation enhancer. As a consequence, binary ethosomes have attracted a lot of attention. The benefits of binary ethosomes compared to traditional ethosomes and transferosomes were reported [135]. It was found that terbinafine-loaded binary ethosomes with an ethanol and propylene glycol ratio of 7:3 (*w*/*w*) displayed substantial enhancement in skin permeation as well as greater rhodamine B fluorescence intensity. Similarly, binary ethosomal gel formulations of triamcinolone displayed a better zeta potential, increased entrapment efficiency and better epidermal penetration than traditional ethosomal gels [136]. In addition, binary ethosomes allow effective drug encapsulation, controlled skin penetration and extended drug retention, which makes them suitable for the delivery of both hydrophilic and lipophilic drugs [137]. As a result, binary ethosomes are appropriate for both localized dermatological applications and systemic delivery through the transdermal route.

Composite ethosomes are produced using a specific combination of saturated and unsaturated phospholipids, like hydrogenated lecithin, PC and soybean lecithin. Within the lipid bilayer, saturated and unsaturated phospholipids interact synergistically to improve the stability of composite ethosomes. Unsaturated phospholipids enhance membrane fluidity, although vesicle stability could be compromised by oxidative breakdown of their double bonds [138]. Integrating saturated phospholipids, which are closely packed and have oxidation-resistant acyl chains, reduces this effect through improving membrane rigidity and oxidative resistance [139]. Meanwhile, unsaturated phospholipids inhibit excessive crystallization of saturated lipids, providing the perfect balance of rigidity and flexibility needed for ethosomal deformability and transdermal penetration. A curcumin-loaded composite ethosomal system developed using a 1:1 ratio of PC and hydrogenated PC showed higher vesicle stability, flexibility and transdermal delivery. This is because of enhanced bilayer stabilization and reduced lipid peroxidation, emphasizing the possibility of delivering poorly stable bioactive compounds [140]. The heterogeneous lipid packing in composite ethosomes enhances resistance to bilayer disruption, which causes improved physicochemical stability and drug retention when compared to regular ethosomes. Composite ethosomes were developed to enhance transdermal delivery of poorly water-soluble ketoprofen [141]. The formulation developed using a single-step injection approach showed good drug loading with improved solubility. Ex vivo skin permeation experiments revealed sustained drug release as well as significant transdermal penetration, with a cumulative permeation of 602.35 ± 41.06 μg/cm^2^. Moreover, composite ethosomes improved drug retention within the skin, facilitating reservoir formation and prolonging drug availability.

Active targeted ethosomes are a better version of ethosomal drug delivery systems developed to provide site-specific delivery through incorporating targeting ligands on the outer layer of ethosomal vesicles [119]. Active targeting enables selective proximity to specific cells, receptors, or diseased tissues due to the natural penetration-enhancing properties of ethanol-rich ethosomes. This in turn can improve therapeutic efficacy while minimizing off-target consequences. Active targeting can be accomplished by modifying the ethosomes surface with specific ligands like sugars, aptamers, peptides, antibodies, and small molecules. Traditional ethosomes can be coated using polymers and functional agents like sodium cholate, polyethyleneimine, hyaluronic acid and galactosylated chitosan. Target cells overexpressing receptors, including CD44, transferrin receptors, or integrins, can be specifically identified and linked in these surface-modified ethosomes. For instance, sodium cholate-modified ethosomes and polyethylenimine-modified ethosomes have been developed and linked through electrostatic interactions to enhance drug delivery [142]. In psoriatic skin, hyaluronic acid surface alteration of ethosomes enhanced curcumin retention and anti-inflammatory effects by enabling CD44-targeted distribution [143]. Similarly, hyaluronic acid surface modification of ethosomes improved cellular absorption of paclitaxel, transdermal penetration and anticancer activity, highlighting the significance of surface engineering in targeted transdermal drug delivery [144]. Surface-modified ethosomes are therefore beneficial for the management of dermatological conditions like wound healing, wherein receptor expression is upregulated.

#### 7.1.2. Composition

The components used in the preparation of ethosomes are summarized in Table 2. Ethosomes are primarily composed of phospholipids with a relatively high concentration of ethanol (~20–45%). The role of phospholipids is to form a stable and rigid vesicular bilayer, whereas alcohol increases bilayer fluidity and vesicle flexibility and enhances skin permeation by disrupting SC lipids. Ethosomes may also include optional additives such as propylene glycol or terpenes as penetration enhancers to further improve drug transport and cholesterol as a membrane stabilizer to reduce leakage. For topical applications, ethosomal suspensions are often converted into ethosomal gels using polymers (gelling agents) along with pH adjusters to improve skin compatibility and residence time. Cryoprotectants may be added to prevent vesicle aggregation during storage.

#### 7.1.3. Wound Healing Applications

In wound healing applications, ethosomes have shown considerable potential by improving the localized delivery of antibiotics, anti-inflammatory agents, antioxidants, growth factors and phytochemicals. Greater dermal penetration results in higher drug accumulation at the wound site, which promotes fibroblast proliferation, collagen deposition, angiogenesis and re-epithelialization. Ethosomal preparations additionally provide extended drug release and improved stability of encapsulated drugs, which is especially helpful in chronic and infected wounds such as diabetic ulcers and burns. Ethosomes encapsulating phytochemicals and synthetic drugs are getting much attention in wound healing. When phytochemicals are integrated into ethosomes, these vesicles can overcome their low solubility and poor skin penetration, which leads to higher bioavailability at the wound site and faster healing. Furthermore, the use of ethosome-based delivery systems containing synthetic drugs and phytochemicals is an interesting approach for wound healing. They improve patient compliance, accelerate epithelialization, regulate infection and minimize inflammation. Thus, this technique is an innovative and successful topical drug delivery approach for acute and chronic wounds.

Metformin-loaded ethosomes formulated with 30% *v*/*v* ethanol exhibited excellent permeability in mouse skin (85.8 ± 3.7) as well as improved drug entrapment (55.3 ± 0.07) [145]. As compared to conventional metformin gel, the carbomer-based ethosomal gel exhibited substantially higher skin permeation (*p* < 0.001). Drug-loaded ethosomes displayed reduced tumor cell viability (*p* < 0.05) and enhanced antiproliferative activity against melanoma B16 cells, resulting in lower IC_50_ values (56.45 ± 1.47 µg/mL), as compared to metformin solution (887.3 ± 23.2 µg/mL). An in vivo study indicated that the wound healing was superior (80.5 ± 1.9%) to that of Mebo^®^ ointment (56 ± 1%, *p* < 0.05). Histopathological data showed that the ethosomal gel greatly enhanced the mRNA expression of important growth factors involved in the healing process (IGF-1, FGF-1, PDGF-B and TGF-β), emphasizing its potential as an effective strategy for the treatment of melanoma and wound healing.

The low bioavailability and poor water-solubility of curcumin have limited its therapeutic use, regardless of its well-established capacity to heal wounds. Its therapeutic efficacy is reduced by its rapid metabolism and systemic elimination [146]. A once-daily topical application of 0.2% ethosomal curcumin formulation (Etho-cur) for 14 days greatly enhanced essential wound healing parameters like granulation tissue growth, collagen deposition, neovascularization and re-epithelialization (*p* < 0.01–0.001) compared to the control [147]. Additionally, Etho-cur accelerated wound contraction, leading to complete wound closure by day 16 (*p* < 0.001). Moreover, Pseudomonas aeruginosa as well as other burn-associated bacterial flora were effectively suppressed by Etho-cur, which showed antibacterial activity similar to 1% silver sulfadiazine cream. Notably, compared to free curcumin, Etho-cur exhibited almost 11% higher antibacterial efficacy.

Several physical methods are being used to improve drug permeation through the skin surface, including iontophoresis [148]. A comprehensive review underlined the use of nanocarrier systems in conjunction with physical enhancement strategies to enhance percutaneous drug penetration [149]. Iontophoresis in conjunction with ethosomal formulations has been suggested as an effective way to enhance transdermal drug delivery. A study has been carried out to evaluate the ex vivo transdermal delivery of ethosomes loaded with hydrocortisone 17-butyrate (HB17) in conjunction with iontophoresis [150]. The negatively charged ethosomal-HB17 exhibited a mean particle size of 244 ± 4.3 nm, an entrapment efficiency of 40.6 ± 2.21% and good stability for up to 30 days. Passive permeation studies showed no drug diffusion from free H17B, while ethosomal H17B showed 7.98 μg/cm^2^ in 120 min. When iontophoresis was applied, the permeation values increased to 19.69 μg/cm^2^ in 30 min for the ethosomal H17B formulation. Overall, the results demonstrate that ethosomal-H17B markedly enhances transdermal drug delivery and that this effect is further amplified when combined with iontophoresis.

In another study, nanoethosomal piroxicam (<200 nm) was developed for transdermal delivery and evaluated in combination with iontophoresis [151]. Although the lecithin content mostly influenced turbidity and pH, the lecithin concentration of 5 mg led to the smallest particle sizes. When the concentration was between 4 and 5.5, entrapment efficiency declined linearly with increasing pH, emphasizing the significance of optimum physicochemical properties over particle size. Indeed, the iontophoresis considerably improved the permeability of the nanoethosomal formulation compared to free and nanoethosomal piroxicams.

Hypertrophic scars are raised, thickened scars that result from abnormal wound healing characterized by excessive fibroblast proliferation and collagen overproduction [152]. They remain confined to the original wound margins and are often associated with symptoms such as pain, pruritus, restricted mobility and psychological distress. Intralesional therapy combining 5-fluorouracil and triamcinolone acetonide is considered a first-line treatment due to its anti-inflammatory and antifibrotic effects [153]. However, the treatment is often painful, particularly due to the dense nature of hypertrophic scar tissue, and may be poorly tolerated by patients. Repeated injections are usually required, increasing discomfort and reducing patient compliance [154]. To address these challenges, ethosomes co-loaded with 5-fluorouracil and triamcinolone acetonide were embedded in a gelatin-oxidized tragacanth gum polymeric film to form a nanocomposite system [155]. The nanoparticles exhibited nanoscale size, high drug encapsulation efficiency, sustained drug release and effective penetration through dense scar tissue. Preclinical studies and a 12-week clinical evaluation demonstrated reduced fibroblast proliferation, improved collagen organization, significant scar improvement and good safety. These observations established the potential of the nanocomposite system as a promising noninvasive therapeutic approach for hypertrophic scars. Table 3 summarizes key formulation characteristics and therapeutic outcomes of ethosomal formulations investigated for wound healing applications.

### 7.2. Transferosomes

Transferosomes are very flexible and ultradeformable lipid-based vesicular carriers developed particularly to overcome the SC [160]. They are structurally made up of an aqueous core surrounded by a phospholipid bilayer modified with edge activators to provide elasticity and enhanced deformability. This unique deformability allows transferosomes to transport drugs, including hydrophilic and even macromolecular compounds like peptides and proteins, through intact skin more effectively than regular liposomes and other vesicles [161]. Additionally, they utilize lipid membrane flexibility to enhance skin permeation and bioavailability and control drug release, which results in better patient compliance and treatment outcomes. It has also been reported that transferosomes can move through the narrow pores of the densely packed SC because of their high elasticity and flexibility [162]. In general, they can travel through the SC as intact vesicles as long as their diameter is smaller than 300 nm. Moreover, transferosomes are well known to enhance drug permeation and provide therapeutic doses similar to those obtained with subcutaneous injection [163]. Various properties of transferosomes are summarized in Table 1. These vesicles generally do not contain ethanol.

According to one hypothesis, the higher percutaneous permeation of transferosomes across the SC is due to the osmotic gradient generated by the differences in water content between the skin surface and deeper layers [164]. Transferosomes were shown to be transported across the SC through this osmotic force; however, penetration into deeper skin layers was not observed in some cases. In nonocclusive settings, they rely on the transepidermal osmotic gradient as the principal driving force, permitting elastic deformation and allowing transport through the intercellular or transcellular routes [165,166]. Nonocclusive application enables water to evaporate from the skin surface and generates a hydration gradient that supports vesicle movement. Experimental data showed that >50% of topically applied tritium-labelled phospholipid (^3^H-DPPC) permeated to deeper skin under nonocclusive conditions [164]. However, ~87% was found to reside at the very surface of the SC under occlusion. Accordingly, transferosomes are likely to permeate the skin through two water-filled paths, namely, the intercluster pathways (between cell groups) and intercellular spaces (between individual corneocytes).

Transferosomal systems are most appropriate for less permeable drugs requiring deep tissue or systemic distribution. They encapsulate the active chemical either within their aqueous core or integrated in the lipid bilayer, depending on its solubility. This property provides an important advantage by facilitating the simultaneous delivery of different drugs and phytochemicals into the systemic circulation [166]. The specific composition and mechanical characteristics of transferosomes allow them to be especially beneficial for the noninvasive delivery of peptides and vaccines into and through the skin, with applications ranging from anti-inflammatory and anticancer drugs to peptides and macromolecules. They surpass liposomes in transdermal delivery because they permeate intact skin and attain higher drug concentrations in deeper layers.

In accordance with electron microscopy data, transferosomes have an irregular, mainly oval shape as edge activators destabilize the bilayer. This provides them with more elasticity and deformability than conventional liposomes, which enhances transdermal penetration [167]. The zeta potential and colloidal stability of transferosomes generally indicate greater physical stability in liquid media than liposomes and niosomes [168]. They are stable without noticeable aggregation for up to three months at both ambient temperature (25 °C) and refrigerated temperature (4 °C).

Regardless of their benefits, transferosomes have certain limitations that hinder their successful translation into general clinical use. Their physical and chemical instability is an important concern since edge activators may destabilize the phospholipid bilayer, which may result in vesicle fusion, aggregation and drug leakage during storage. Increased membrane fluidity and reduced entrapment efficiency are the consequences of greater levels of edge activators, which also induce pore formation, disrupt bilayer organization and increase membrane deformability [169]. On the other hand, lower edge activator levels could result in larger vesicle size [170]. Higher surfactant concentrations can change vesicle shape, with levels beyond the threshold encouraging micelle production and increasing vesicle size [171]. Moreover, excessive amounts may impair drug entrapment and penetration, as indicated by the decreased cilnidipine flux at greater surfactant levels [172]. There has been no clinical translation of transferosomes, and regulatory challenges regarding their long-term safety, stability and quality control have yet to be resolved [173].

In comparison to liposomes, these vesicles show lower loading efficiency with lipophilic drugs due to their hydrophilic surface. Furthermore, the hydration gradient across human skin is nonlinear, with areas near the viable epidermis showing lower moisture than the central SC. Unequal hydration may reduce the osmotic gradient, which in turn leads to the formation of drug depots in the skin [174]. Strict storage conditions are necessary to maintain the integrity of transferosomes as they are naturally susceptible to oxidative degradation and temperature changes. This vulnerability is mainly due to the hydroxyl functional groups within phospholipid fatty acid moieties, which encourage lipid oxidation and impair formulation stability [175]. Formulation stability and industrial scalability remain a challenge in manufacturing. This is because traditional methods of preparation such as thin-film hydration and sonication are difficult to scale up while maintaining identical vesicle size and deformability [176]. Additionally, the use of surfactants or bile salts as edge activators could cause skin irritation or toxicity after continuous usage, and variations in skin permeability might cause uneven drug absorption [163]. Diractin^®^ is the only marketed transferosomal formulation; nevertheless, it was withdrawn shortly after approval due to its minimal clinical effect relative to traditional ketoprofen gels [177].

#### 7.2.1. Composition

Transferosomes are mainly composed of phospholipids, which compose the bilayer membrane, provide structural integrity, and enable drug encapsulation and interaction with skin lipids (Table 2). Edge activators provide elasticity and deformability, allowing vesicles to navigate through the SC. Typically, organic solvents are used to dissolve lipids, which are then hydrated with an aqueous phase that promotes vesicle formation and regulates pH, size and stability. In addition, excipients like chemical skin enhancers are included to temporarily disrupt skin lipid packing and improve permeation. Similarly, they may include membrane stabilizers to reduce leakage, charge-inducing agents to tune surface charge, and cryoprotectants for freeze-drying and storage protection.

#### 7.2.2. Wound Healing Applications

Transferosomes are a promising vesicular drug delivery approach for wound healing due to their greater skin penetration as well as higher drug loading capacity [178]. Their specific composition allows the breakdown of the SC lipid structure, improved drug penetration and sustained drug release at the wound site. Transferosomes are frequently used to load synthetic drugs and natural components, leading to improved skin penetration, extended release and greater therapeutic efficacy in wound healing applications. This type of vesicle has also been widely investigated for the topical delivery of antimicrobial agents, anti-inflammatory drugs, growth factors, and antioxidants. This effort improved wound closure rates, decreased infection, increased collagen deposition, and expedited tissue regeneration.

Diabetic nonhealing ulcers, especially pressure ulcers and diabetic foot ulcers, provide a significant treatment challenge and are the main cause of non-traumatic amputations [179]. They are caused by poor wound healing mechanisms linked to chronic hyperglycemia, such as impaired angiogenesis, neuropathy, ischemia, prolonged inflammation and increased susceptibility to infection. These characteristics promote delayed epithelialization, high oxidative stress and poor collagen remodeling, making diabetic wounds difficult to treat and prone to chronicity and recurrence. Current treatment alternatives, such as becaplermin gel, have been linked to safety concerns, including increased risk of cancer, emphasizing the critical need for safer and more effective treatment solutions. Hyperglycemia significantly reduces the activity of HIF-1α, which regulates angiogenesis, cell proliferation, migration and survival during wound healing [180]. Deferoxamine, an iron chelator, has shown promise in the treatment of diabetic wounds by stabilizing HIF-1α through the inhibition of prolyl hydroxylase enzymes. This large hydrophilic drug was delivered to the skin more effectively by using transferosomes as nanocarriers, which increased transdermal penetration and enabled continuous release [181]. The optimized iron chelator-loaded transferosomal gel exhibited high entrapment efficiency, nanoscale vesicle size, appropriate surface charge, uniform drug distribution, and controlled release and demonstrated enhanced wound healing in a diabetic ulcer model, highlighting its potential for treating chronic diabetic ulcers.

Transferosomes encapsulating phytochemicals have been explored for wound healing because many plant bioactives suffer from poor aqueous solubility, low skin permeability and chemical instability when applied as conventional creams/gels [182]. Transferosomes can enhance deposition into deeper skin layers and provide localized, sustained release, which is particularly useful for chronic wounds where prolonged antioxidant/anti-inflammatory and antimicrobial action is desired. In wound healing models, phytochemical-loaded vesicular systems have been repeatedly linked to improved outcomes such as faster re-epithelialization, reduced inflammation/oxidative stress and better collagen organization. For example, curcumin has been successfully incorporated into transferosomal gels with reported wound healing potential, supporting the concept that transferosome encapsulation can improve the performance of poorly permeable phytochemicals in topical therapy [183].

Burn wounds are complex skin injuries that significantly impair normal skin function and cellular activity, both of which are essential for effective healing. Extensive skin damage necessitates the use of biocompatible dressings that not only protect the wound but also support the natural repair process. Fusidic acid is an antibiotic obtained from the fermentation of *Fusidium coccineum*. It is widely used in the treatment of staphylococcal infections, impetigo, infected dermatitis and contaminated cuts and wounds by inhibiting bacterial protein synthesis through interference with translocation. Fusidic acid-loaded transferosomal gel is therefore suggested to be useful in burn wound management by preventing or reducing the risk of secondary bacterial infections [184]. A transferosomal lidocaine gel was developed using HPMC K15 as the gelling agent and propylene glycol, dimethyl sulfoxide and polyamidoamine G3 dendrimer as permeation enhancers. The optimized formulation showed ideal characteristics with good entrapment efficiency (79.87 ± 2.35%), a small particle size (179.5 nm) and a high negative zeta potential (−43.5 ± 4.74 mV). The transferosomal gel containing polyamidoamine G3 demonstrated significantly enhanced analgesic activity in the tail flick test, with a 1.62-fold increase in AUC_0–90_ compared to the control lidocaine solution. These results imply that the new formulation may be a promising topical therapy for painful disorders like burns, which offers efficient local anesthesia without the need for injections. Mangiferin possesses a variety of medicinal properties, including antioxidant, anti-inflammatory, antimicrobial and wound healing effects; however, its therapeutic potential is restricted by its poor aqueous solubility, low lipophilicity, rapid clearance and poor skin penetration [185]. Incorporation into modified transferosomal vesicles enhances its dermal and transdermal delivery. Excipients like glycerol, propylene glycol and mucin enhance skin hydration, penetration, vesicle stability and mucoadhesion, resulting in improved skin retention and bioavailability [186].

Applications for wound healing and anti-aging frequently use recombinant human epidermal growth factor (rhEGF) [187]. RhEGF facilitates faster and more effective wound healing by increasing angiogenesis, tissue regeneration and cell proliferation, which are key biological processes that are frequently interrupted in such lesions. However, its fast degradation by proteolytic enzymes and restricted skin penetration limit its topical efficiency. A study has been conducted to enhance the skin distribution of recombinant human epidermal growth factor using conventional liposomes (rhEGF-CLs) and transferosomes (rhEGF-TFs) [188]. rhEGF-TFs with 0.05–1.0 μg/mL rhEGF showed ideal characteristics due to favorable release patterns, suitable encapsulation efficiency, greater cell proliferation and minimal cytotoxicity, while higher levels reduced cell viability. The optimized formulation (rhEGF-TFs-2) with a tween 80: lipid ratio of 20:80 had a particle size of 233.23 ± 2.64 nm, a PDI of 0.33 ± 0.05, a zeta potential of −15.46 ± 0.29 mV and an EE% of 60.50 ± 1.91 and was found to be stable at 5 °C for one month. When compared to free rhEGF, the formulation showed a unilamellar structure, constant rhEGF release (~82% within 24 h) and substantially higher dermal penetration, suggesting its potential for use in wound healing and skin regeneration.

Pathological manifestations of abnormal wound healing, including hypertrophic scars and keloids, are caused by deregulation of the normal repair process [189]. Hypertrophic scars and keloids are caused by prolonged fibroblast activation, excessive collagen synthesis and inadequate collagen degradation, while natural wound healing involves tightly regulated phases of inflammation, proliferation and remodeling. Although keloids spread beyond the original injury site and may continue to grow over time, hypertrophic scars are confined to the original wound margins. These conditions are linked to chronic inflammation, increased ECM deposition and altered growth factor signaling [190]. To obtain deformable vesicular systems suitable for topical application, papain-loaded transferosomes have been developed by the thin-film hydration technique using soy lecithin as the phospholipid, tween 80 as the edge activator and cholesterol as the membrane stabilizer [191]. These transferosomes present a viable noninvasive treatment option for hypertrophic scars and keloids by enabling efficient papain distribution through the epidermal barrier. In contrast to papain solution and traditional liposomes, papain-loaded transferosomes effectively penetrated the SC and exhibited preferential deposition within the epidermal and dermal layers without penetrating full-thickness skin, which is useful in localized scar therapy. Papain encapsulation in transferosomes also maintained cell viability at low doses and decreased the epidermal damage observed with free papain, indicating better safety. The potential of papain-loaded transferosomes as a topical scar management approach has been demonstrated by their higher skin penetration, controlled localization and improved biocompatibility.

It has been reported that the human growth hormone (hGH) improves wound healing by promoting fibroblast proliferation, collagen synthesis, ECM remodeling and angiogenesis [192,193]. Dermal fibroblasts act as key effector cells in the proliferative and remodeling phases of wound healing, and their activity has a direct effect on wound closure and tissue strength. However, topical delivery of hGH can be challenging owing to its large molecular size, instability and low skin permeability. Transferosomes significantly enhance the transdermal and dermal delivery of macromolecules like hGH due to their ultradeformable vesicular structure. Dermal fibroblasts can effectively deliver hGH when it is transported by transferosomes, resulting in improved matrix organization, greater collagen deposition and cellular proliferation. The potential of hGH-loaded transferosomes (F1 and F2) in transdermal therapy has been examined [194]. It was found that fibroblast migration, proliferation, and collagen type I and III gene expression were considerably enhanced following transferosome-mediated hGH delivery. Skin permeation data showed that F1 was a more effective and nontoxic carrier, delivering more hGH (489.54 ng/cm^3^ over 24 h) than F2 (248.46 ng/cm^3^). Furthermore, effective amounts of encapsulated hGH promoted fibroblast growth and production of collagen, with a substantial increase in cell migration detected at 700 ng/mL, when compared to the control.

Overall, transferosome-based formulations improve the topical delivery and efficacy of synthetic drugs, phytochemicals and macromolecules like hGH and rhEGF by overcoming skin permeability and stability barriers. Transferosomes are a promising noninvasive method for treating acute and chronic wounds as they promote essential wound healing processes such as fibroblast activity, collagen formation and angiogenesis while reducing systemic side effects. Table 4 presents a mini-database summarizing the composition, functional performance, and wound healing outcomes of transferosome-based gel formulations.

### 7.3. Transethosomes

These are ultradeformable lipid vesicular carriers developed to enhance dermal and transdermal drug delivery by combining the advantages of ethosomes and transferosomes. They are typically composed of phospholipids and high concentrations of ethanol and edge activators such as tween, span, or bile salts. Various properties of transethosomes are summarized in Table 1. The presence of ethanol and edge activators in the composition allows transethosomes to enter deeper skin layers with greater efficiency than conventional liposomes, ethosomes, or transferosomes. The higher skin permeation of transethosomes is due to various mechanisms functioning together. Ethanol alters and fluidizes the SC lipid structure, reducing barrier resistance, while the highly flexible vesicular membrane allows transethosomes to move through pores that are substantially smaller than their own diameter without rupturing [200]. Furthermore, vesicle movement into deeper skin layers is assisted by the moisture gradient resulting from topical therapy. Transethosomes are particularly suitable for targeted and persistent drug delivery because these processes additionally enhance transdermal flux and promote higher drug deposition in the viable epidermis and dermis. Sinapic acid is a bioactive phenolic molecule with limited aqueous solubility; hence, it shows low therapeutic efficacy. In one study, the transdermal flux of this bioactive from transethosomes was found to be considerably greater (2.93 ± 0.16 µg/cm^2^/h) than that of the free drug (1.03 ± 0.07 µg/cm^2^/h) [201]. This enhanced efficiency is attributed to the deformability of transethosomes, which enables deeper skin transport and increased drug distribution across the membrane. Furthermore, this vesicular system showed less skin irritation and is described as being suitable for delivering drugs with a wide range of molecular weights [202]. The low viscosity and poor skin retention of transethosomes made it imperative to include them in gel systems. Transethosomal gels prolong the duration of residency on the skin while successfully encapsulating unstable, high-molecular weight herbal extracts and bioactive substances, which facilitates deeper skin penetration [203]. Various advantages of transethosomal gels include improved patient compliance, bypassing first-pass metabolism, increased bioavailability, high drug entrapment efficiency and controlled drug release.

Transethosomes do have certain limitations like ethosomes that must be addressed before they can be utilized in clinical situations. They have a more complex composition with high alcohol and surfactant contents and a soft bilayer structure. This could lead to physical and colloidal instability, causing vesicle aggregation, fusion, or drug leakage while storing. Ethanol and some excipients could lead to skin irritation or dermatitis in vulnerable people. In addition, attaining uniform particle size, deformability and encapsulation efficiency on a large production scale is difficult, complicating reproducibility and regulatory compliance. These limitations emphasize the importance of careful optimization of formulation factors along with stabilizing techniques to ensure product shelf life and patient safety [204].

#### 7.3.1. Composition

The components used in the preparation of transethosomes are summarized in Table 2. Transethosomes are made up of a phospholipid bilayer, alcohol and edge activators. The phospholipid allows both drug entrapment and interaction with SC lipids, with hydrogenated/high-transition temperature lipids increasing rigidity and stability. Alcohol enhances drug solubility, bilayer fluidity and skin permeability. The edge activators provide ultradeformability for passage through small skin routes. An aqueous phase hydrates vesicles and influences pH/stability, while optional penetration enhancers and cholesterol may further improve permeation and reduce leakage. For topical use, transethosomes may be incorporated into gels with suitable pH adjustment, and cryoprotectants can be added to maintain vesicle integrity during storage.

#### 7.3.2. Wound Healing Applications

Transethosomes are useful carriers in wound healing for both synthetic drugs and phytochemicals. When loaded with synthetic agents, they improve dermal deposition and provide sustained release at the wound site to reduce microbial burden, limit excessive inflammation and support progression to the proliferative phase. When loaded with plant-derived bioactives (polyphenols, flavonoids, terpenoids, etc.), they deliver compounds with antioxidant, antimicrobial and anti-inflammatory actions that are important for chronic wounds. Transethosomes are nanoscale products with a high entrapment efficiency and are frequently integrated into hydrogels, films, or nanofiber dressings to extend residence time and maintain a moist environment, resulting in faster wound closure by improved re-epithelialization, fibroblast activity, collagen deposition/remodeling and angiogenesis, while possibly decreasing systemic side effects.

**Figure 2:**
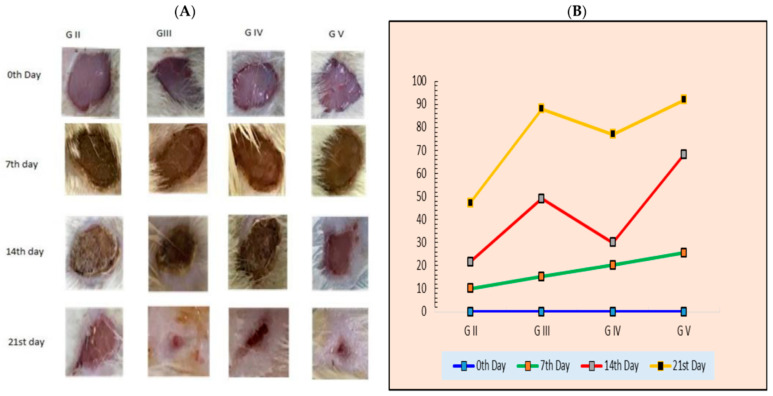
Representative photographs (**A**,**B**) illustrating wound healing progression at different time intervals (0th day, 7th day, 14th day, 21st day) across the various experimental groups (Group II: wounded animals receiving no treatment, Group III: wounded animals treated with silver sulphadiazine ointment (1% *w*/*w*), Group IV: wounded animals treated with a drug-loaded gel containing 1% ROS in 2.5% HPMC K4M, Group V: wounded animals treated with the optimized ROS-loaded transethosomal gel formulation). Reproduced from Ref. [208].

Statins have shown efficacy in the treatment of a wide variety of dermatologic disorders, such as urticaria, psoriasis, and acne, as well as being useful in wound healing. Rosuvastatin can increase wound healing by neutralizing inhibitors such as farnesyl pyrophosphate and improving microvascular and endothelial function [205]. It also exhibits antibacterial effects by disrupting bacterial protein synthesis and multiple cellular/biosynthetic pathways. This can inhibit production of methicillin-resistant *Staphylococcus aureus* (MRSA) toxins that contribute to delayed progression of septic skin lesions [206]. However, rosuvastatin efficacy is limited by poor aqueous solubility and low oral bioavailability (<20%) [207], making topical delivery an attractive alternative. An optimal rosuvastatin transethosomal formulation prepared with span 60 (0.819439% *w*/*v*), ethanol (40% *w*/*v*) and lecithin (100 mg) showed ~66.55% entrapment efficiency, an ~277.7 nm vesicle size and a zeta potential of −33 mV [208]. The product exhibited higher drug release than the drug suspension and was stable for one month. Wound healing was evaluated by measuring wound diameter for 21 days, with the untreated wounded group (Group I) serving as the control. All groups showed a progressive reduction in wound area compared with the baseline; however, wound epithelization was delayed in the untreated group (Figure 2A,B). Among all treatments, Group V, treated with the ROS-loaded transethosomal gel, demonstrated the greatest wound closure and superior healing compared with other groups. This effect is attributed to improved skin penetration of nanosized transethosomal vesicles facilitated by the presence of edge activators and ethanol when compared with the standard silver sulphadiazine ointment (1% *w*/*w*). Statistical analysis confirmed significant differences in wound healing among all groups and across days 7, 14, and 21 (*p* < 0.05), supporting the superior wound healing performance of the transethosomal formulation over the untreated control.

In a comparable study, an atorvastatin-loaded transferosome formulation exhibited a particle size of 238.0 ± 6.65 nm, a PDI of 0.163 ± 0.069, a zeta potential of −45.2 ± 0.07 mV and an encapsulation efficiency of 86.8 ± 3.01% [209]. The corresponding drug-loaded transferosomal gel showed suitable rheological performance, with a viscosity of 5657 ± 127.27 cP and a spreadability of 300 ± 50%. Both transferosome and its gel demonstrated sustained drug release, achieving 92.60% and 78.45% of atorvastatin release at pH 7.4 after 24 h, respectively. Skin permeation was also significantly enhanced, as the transferosome (15.67 μg/cm^2^/h) and drug-loaded gel (11.67 μg/cm^2^/h) produced a markedly higher flux than the control (atorvastatin–propylene glycol, 4.21 μg/cm^2^/h). In a mouse excision wound model, the gel produced considerably higher wound closure (92.5% ± 1.5) than in the control (62.8% ± 3.2) or the untreated group (48.9% ± 4.0), suggesting better healing. These findings were confirmed by histological analysis, which indicated that transferosome gel-treated skin had enhanced dermal architecture, collagen organization and re-epithelialization when compared with the other groups.

To improve the efficacy of curcumin, a drug-loaded transethosome was developed using the thin-film hydration technique [210]. The formulation achieved good drug loading (99.7%), a low PDI (0.127 ± 0.022) and a small particle size (115.8 ± 4.9 nm). It was noticed that the curcumin deposition was much higher than in the control (*p* < 0.05) in all skin layers, while a very limited amount of drug reached the systemic circulation. The greater drug penetration was confirmed by FTIR, which showed lipid breakdown in the SC. Compared to free curcumin, the formulation was less cytotoxic and maintained its antibacterial activity against *Staphylococcus aureus* (IC_50_: 12.76 µg/mL vs. 6.2 µg/mL). Wound closure was about 25% quicker and completely repaired in 72 h. Overall, transethosomes showed great potential for wound treatment by improving curcumin skin targeting, lowering toxicity and accelerating the process of in vitro wound healing.

In brief, transethosomes are better than conventional ethosomes at healing wounds because they combine ethanol-driven fluidization of the SC with the additional deformability provided by edge activators. These vesicles are more malleable, stable under physiological stress and are capable of crossing the damaged skin, resulting in more uniform drug deposition within the wound bed and surrounding tissue. Consequently, they improve local drug retention, which is especially beneficial for antimicrobials, anti-inflammatories, antioxidants and growth promoters. In general, transethosomes are superior to ethosomes in terms of permeability, cargo delivery efficiency and therapeutic residence time at the target site, making it possible to accelerate key wound healing phases.

### 7.4. Preparation Methods

Ultradeformable vesicles are commonly prepared using lipid self-assembly approaches followed by a size-reduction step to obtain nanoscale, uniform dispersions. Thin-film hydration, the hot method, the cold method and ethanol injection are among the most frequently used techniques for preparing ultradeformable vesicles. In addition, several miscellaneous approaches are also employed, including reverse-phase evaporation, the modified shake flask method, vortexing/shaking methods and microfluidic techniques.

#### 7.4.1. Thin-Film Hydration Method

**Figure 3:**
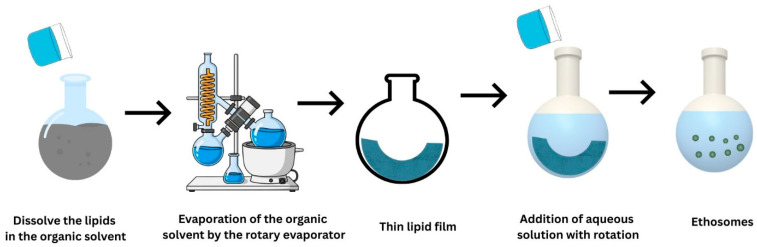
Schematic diagram illustrating the thin-film hydration method.

Thin-film hydration (rotary evaporation) followed by size reduction is a common method used to prepare transferosomes, transethosomes and some ethosome variants [126]. In this approach, the phospholipids and drug are first dissolved in a volatile organic solvent. For transferosomes and transethosomes, an edge activator/surfactant is included. The solvent is then removed under reduced pressure using a rotary evaporator to form a uniform thin lipid film on the flask wall (Figure 3). Nitrogen gas is flushed through the flask, which is then kept at room temperature for 24 h to ensure complete removal of residual solvent. This film is subsequently hydrated with an aqueous phase (e.g., phosphate-buffered saline, distilled water, or buffer. For ethosomes/transethosomes, hydroethanolic media such as 10–40% *v*/*v* ethanol are used to generate vesicular dispersions. The hydration medium is typically maintained above the lipid gel at liquid crystalline transition temperature to increase bilayer fluidity and optimize lipid/edge activator packing, which can affect vesicle size distribution and morphology [211]. Finally, the dispersion is size-reduced and homogenized using sonication (bath or probe sonication) and/or membrane extrusion (e.g., polycarbonate membranes of 100–400 nm pore size) to produce nanosized vesicles with improved size uniformity and stability. The duration of hydration affects the extent of bilayer swelling and equilibration, which in turn impacts vesicle size distribution and drug partitioning, especially after the size-reduction process [212]. The pH of the hydration medium is also important because it changes drug ionization and solubility, thereby altering drug partitioning between the aqueous phase and the lipid bilayer and ultimately influencing encapsulation efficiency, leakage and formulation stability.

For example, curcumin-loaded transethosomes have been prepared by dissolving phospholipids and surfactant/edge activators. Curcumin was added to an organic solvent and a thin lipid film was formed by rotary evaporation. Hydration of the film was performed with aqueous or hydroethanolic buffer and followed by probe sonication/extrusion to obtain nanosized vesicles [213]. Such curcumin transethosomal systems are commonly reported to enhance cutaneous deposition and provide sustained local release, supporting their potential use in inflammatory or impaired-healing skin. Transferosomal vesicles loaded with meloxicam and dexamethasone were prepared using lecithin along with edge activators/surfactants (span 80 and tween 80) by the thin-film hydration method [214]. The resulting vesicles were size-reduced by sonication or extrusion to obtain more uniform nanosized systems. The prepared transferosomes achieved vesicle sizes of approximately 248–273 nm, with a high negative zeta potential (about −62.6 to −69.5 mV), a moderate polydispersity index (PDI; 0.329–0.526) and substantial entrapment efficiency for both drugs.

#### 7.4.2. Cold Method

**Figure 4:**
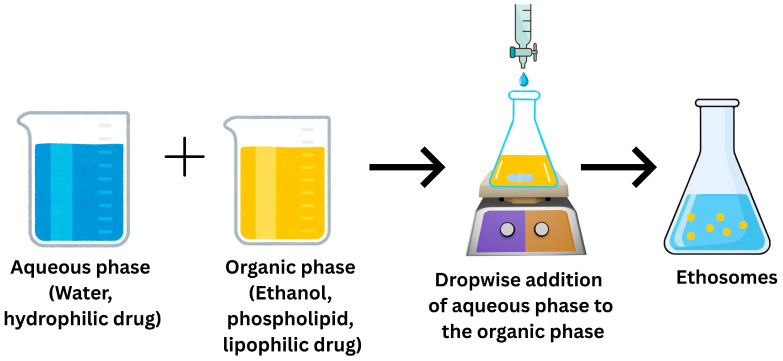
Schematic diagram illustrating the cold method.

The cold method is a commonly reported technique for preparing ethosomes and has been widely adapted for transethosomes, particularly when the drug is thermolabile [126,128]. In this approach, phospholipids are first dissolved in ethanol at room temperature under continuous stirring (typically using ethanol in the ~10–45% *v*/*v* range, depending on the formulation) (Figure 4). For transethosomes, an edge activator is then incorporated to enhance vesicle deformability. A warmed aqueous phase is added slowly to the ethanolic lipid phase under vigorous mixing, leading to spontaneous vesicle formation. The resulting dispersion is then downsized using sonication and/or membrane extrusion to achieve nanoscale vesicles with a narrow size distribution, after which formulations are typically stored under refrigeration to improve their physical stability. Bifonazole transethosomes were prepared using the cold method and optimized via a 3^2^ factorial design to improve skin permeation and antifungal efficacy [215]. The formulations showed high entrapment (66.0 ± 2.64% to 85.1 ± 1.01%), small particle sizes (162 ± 1.30 to 281.9 ± 1.40 nm) and good PDI (<1). The optimized formulation had a size of 162 ± 1.30 nm, a −26.07 mV zeta potential and a 77.6 ± 0.72% EE, with spherical, uniform vesicles observed via TEM.

#### 7.4.3. Hot Method

**Figure 5:**
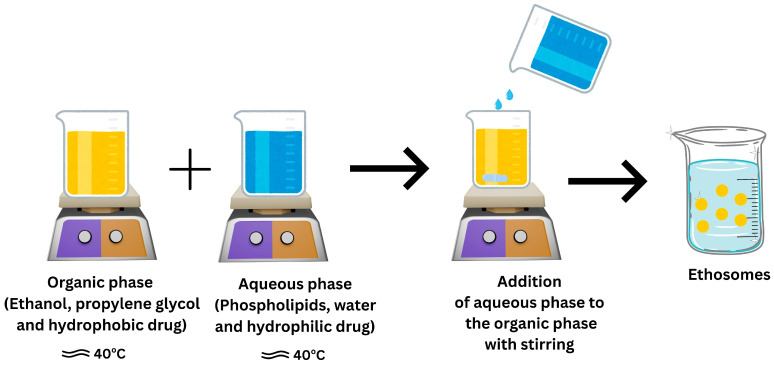
Schematic diagram illustrating the hot method.

This method involves dispersing phospholipids in an aqueous medium and maintaining both phases at the same elevated temperature to promote vesicle formation and uniformity. Briefly, phospholipids such as PC or phosphatidylserine are dispersed in purified water and heated to 40 °C in a water bath until a colloidal solution is formed. In another vessel, a mixture of ethyl alcohol and propylene glycol or glycerol is prepared and kept at the same temperature. Then the warm aqueous phospholipid dispersion is added gradually into this alcohol mixture under continuous stirring for about 7–10 min to obtain a uniform colloidal vesicular system [127]. The drug is dissolved separately either in ethanol or water, depending on its solubility, and this solution is then incorporated into the prepared colloidal mixture while maintaining the process at a constant 40 °C to obtain ethosomes (Figure 5). For transferosomes, the hot method primarily involves hydrating phospholipids with an aqueous phase in the presence of edge activators, typically without high ethanol, to yield ultradeformable vesicles. The dispersion is usually refined to the desired nanosize and uniformity by sonication or membrane extrusion. In a study, Pyrus communis fruit extract was formulated into ethosomes using the hot method and optimized using a central composite design [216]. The optimized ethosomal formulation showed a particle size of 699 nm and a zeta potential of −16.07 mV. Piperine-loaded binary ethosomes were prepared by the hot method to enhance transdermal delivery and subsequently incorporated into a gel [217]. The optimized binary ethosomes exhibited a vesicle size of 187.7 ± 4.63 nm, a PDI of 0.253, a zeta potential of −37.3 mV and an entrapment efficiency of 75.12 ± 0.85%.

#### 7.4.4. Ethanol Injection Method

**Figure 6:**
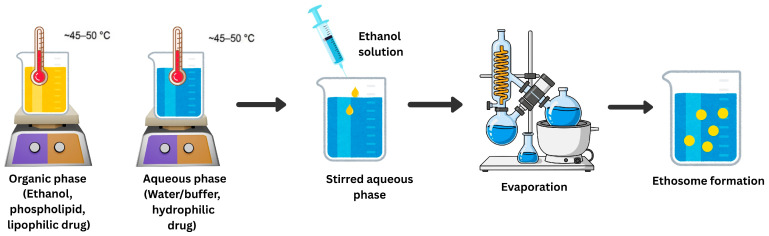
Schematic diagram illustrating the ethanol injection method.

In this method, an ethanolic solution of vesicle-forming components is introduced into a continuously stirred aqueous phase to trigger spontaneous self-assembly. In practice, the organic phase is prepared by dissolving the phospholipid together with an edge activator/surfactant and a lipophilic drug in ethanol until a clear solution is obtained [119]. In parallel, the aqueous phase is prepared by dissolving water-soluble components and a hydrophilic drug in water or phosphate buffer. Both phases are commonly brought to a similar temperature (~45–50 °C), and the ethanolic lipid surfactant solution is then injected dropwise into the aqueous phase under continuous stirring (Figure 6). As ethanol rapidly diffuses into water, the dissolved lipids precipitate into bilayer fragments that fuse and close into vesicles, with the edge activator imparting the characteristic membrane flexibility/deformability. After vesicle formation, ethanol is often reduced/removed by vacuum/rotary evaporation, and the dispersion is typically size-reduced via sonication and/or membrane extrusion to obtain uniform nanosized vesicles suitable for transdermal delivery.

Ginger extract-loaded transethosomes were prepared using the cold injection method by testing different edge activators. The optimized formulation used sodium deoxycholate and produced vesicles with a particle size of 188.3 ± 7.66 nm, a zeta potential of −38.6 ± 0.08 mV and an encapsulation efficiency of 91.0% ± 0.24% [218]. In vitamin D3-loaded transferosomes prepared by the ethanol injection method, the study identified sonication as the primary step controlling the final vesicle size, making it a key parameter for process optimization and scale-up [219]. It also reported a positive linear relationship between surface tension and transferosome size, suggesting that surface tension measurements may help predict vesicle size during processing. The formulation achieved an >90% encapsulation efficiency for vitamin D3, and drug–membrane interactions were highlighted as important for vesicle stability. An ethanol injection method was used to prepare luliconazole-loaded transethosomes to overcome the drug’s low solubility and poor skin permeability [220]. This method produced vesicles of ~246 nm with an ~82% entrapment efficiency and a zeta potential of ~36 mV and improved skin deposition (~35.7%) compared with a luliconazole solution in propylene glycol (~25.3%). However, it was less effective than thin-film hydration in terms of vesicle formation and skin deposition.

#### 7.4.5. Miscellaneous Methods

**Figure 7:**
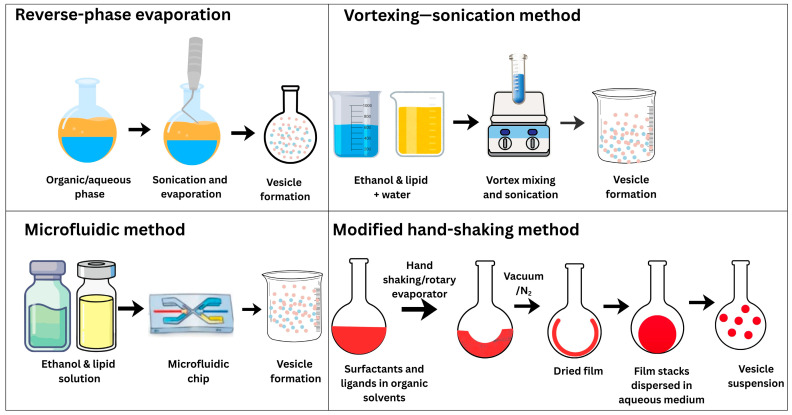
Schematic diagram illustrating miscellaneous methods for the preparation of ultradeformable vesicles.

Several additional techniques beyond the commonly used approaches can be employed to prepare nanovesicular carriers, with the choice of method largely depending on the physicochemical properties of the drug, the desired vesicle size and lamellarity, and the targeted encapsulation efficiency and stability [221]. A schematic representation of the various miscellaneous vesicle preparation methods is presented in Figure 7. Reverse-phase evaporation involves dissolving phospholipids in a suitable nontoxic class 3 organic solvent and combining this lipid phase with a drug-containing aqueous phase under agitation to generate a dispersed system. Gradual solvent removal under reduced pressure causes phase transitions that promote lipid self-assembly into bilayered vesicles dispersed in the aqueous phase. This approach is frequently associated with high encapsulation efficiency for hydrophilic drugs and is followed by size-reduction steps to obtain the desired vesicle size and uniformity. The modified hand-shaking method is a simplified rotary evaporation sonication approach in which lipids and edge activators are dissolved in an organic solvent and mixed with the aqueous drug phase [211]. The mixture is vigorously hand-shaken to form a milky vesicular dispersion, typically followed by sonication or extrusion for size control. In the vortexing sonication method, intense mechanical mixing is used to rapidly disperse lipid/surfactant components into the aqueous medium, and sonication is then applied as the principal energy input to break down larger vesicles into smaller ones [222]. In the microfluidic method, vesicles are prepared using a controlled, injection-like process in which the lipid and aqueous phases are mixed within microscale channels often with varying geometries to achieve highly reproducible vesicles [223]. However, the technique may be restricted by the complexity of microchip design, the possibility of channel clogging and the production of diluted dispersions, which usually require additional concentration [224].

## 8. Evaluations

### 8.1. In Vitro

A comprehensive set of physicochemical, rheological, performance (release/permeation/retention), stability and skin safety tests is typically employed to evaluate ultradeformable vesicles used for wound healing. The main aim of these studies is to ensure vesicle quality, topical usability, dermal delivery and tolerability. Vesicle size and size distribution are generally determined by dynamic light scattering or photon correlation spectroscopy. With a PDI < 0.3, a zeta potential normally ≥±30 mV and a pH of ~5–6.5, an average vesicle size of <500 nm is likely to promote skin compatibility and physical stability [195,211]. Vesicle morphology and aggregation are evaluated using transmission electron microscopy. Drug content, encapsulation efficiency and drug loading are measured by separating the free, unencapsulated drug using ultracentrifugation or ultrafiltration, as well as dialysis techniques. Quantification is carried out using UV spectroscopy or high-performance liquid chromatography to ensure dose uniformity and sufficient payload incorporation [208,209].

Thermal characterization techniques may also be used to evaluate the stability and physicochemical behavior of ultradeformable vesicular systems. Determination of the glass transition temperature (Tg) using differential scanning calorimetry provides information on lipid bilayer phase transitions and membrane fluidity, which can influence vesicle integrity, drug encapsulation, and release behavior [225]. In addition, thermogravimetric analysis measures weight changes with increasing temperature, allowing assessment of thermal stability, solvent loss, and degradation of formulation components [226]. These techniques therefore provide valuable insights into vesicle stability, formulation robustness, and storage conditions for lipid-based nanocarriers such as ethosomes, transferosomes, and transethosomes used in topical wound therapy. Encapsulation efficiency (%EE) and drug loading (%DL) are important parameters used to evaluate the performance of ultradeformable vesicular systems in topical drug delivery. Encapsulation efficiency represents the percentage of the total drug that is successfully entrapped within the vesicular carrier and is calculated as follows:

$$\%EE=\frac{\left[\left(total\;drug-free\;drug\right)\right]}{\left(total\;drug\right)}\times 100$$

A higher %EE indicates efficient incorporation of the drug into the vesicle bilayer or aqueous core, which is essential for maintaining drug stability, preventing premature leakage, and ensuring sustained drug availability at the wound site.

%DL refers to the amount of drug incorporated relative to the total lipid or carrier material and is calculated as follows:

$$\%DL=\left[\frac{encapsulated\;drug}{total\;lipid\left(or\;total\;carrier\;material\right)}\right]\times 100\;where\;encapsulated\;drug=total\;drug-free\;drug$$

This parameter reflects the drug-carrying capacity of the vesicular system and helps determine formulation efficiency and dosing requirements. Ultradeformable vesicles with high encapsulation efficiency and appropriate drug loading can enhance local drug deposition, improve therapeutic efficacy, and reduce the frequency of application in topical wound treatments.

Viscosity/flow behavior, spreadability, adhesiveness and extrudability are evaluated using viscometers/rheometers, as well as texture analyzers, to predict ease of application, skin retention and dose uniformity.

The deformability index (DI) is an important mechanical parameter used to characterize ultradeformable vesicles, reflecting their ability to pass through skin pores smaller than their own diameter [196,227,228]. Deformability is typically evaluated by extruding the vesicle suspension through polycarbonate membranes under controlled pressure and calculating the deformability index using the following relationship:

$$DI=J{\left(\frac{r_{v}}{r_{p}}\right)}^{2}$$

where J represents the amount of vesicle suspension extruded per unit time, $r_{v}$ is the vesicle size after extrusion, and $r_{p}$ is the pore radius of the membrane. A higher deformability index indicates greater membrane elasticity and improved ability of vesicles to penetrate the stratum corneum intercellular pathways. Therefore, deformability measurements provide valuable insight into the mechanical flexibility and penetration potential of ultradeformable vesicular systems used for topical wound therapy.

Lipophilicity is an important factor influencing the skin permeation behavior of drugs delivered through ultradeformable vesicular systems such as ethosomes, transferosomes, and transethosomes. It is commonly expressed by the partition coefficient (P), which describes the distribution of a compound between a lipophilic phase (typically n-octanol) and an aqueous phase. The partition coefficient is calculated as follows:

$$P=\frac{C_{octanol}}{C_{water}}$$

where $C_{octanol}$ and $C_{water}$ represent the equilibrium concentrations of the drug in the octanol and aqueous phases, respectively.

The logarithmic form (log P) is widely used to indicate drug lipophilicity and its potential to partition into the lipid-rich stratum corneum. Drugs with moderate lipophilicity generally exhibit improved skin permeation due to balanced solubility in both lipid and aqueous environments [229].

The functional performance of ultradeformable vesicles for wound therapy is evaluated by drug release, permeation and skin deposition, which assess whether a product delivers a drug topically or into viable tissue while maintaining local availability.

In vitro permeation testing using a Franz diffusion cell fitted with a dialysis membrane is used to generate cumulative release-time profiles and fit various kinetic models [230,231]. The dialysis membrane molecular weight cutoff is typically selected between 12 and 14 kDa to allow the free drug to readily diffuse while retaining the vesicles, with sink conditions maintained in the receptor compartment. Ex vivo permeation is commonly performed using a Franz diffusion cell with excised skin, most often human skin as the closest in vivo surrogate or porcine skin as a practical alternative, while rodent skin may also be used but often leads to overestimation of permeability [232]. Membranes may be full-thickness, split-thickness, or dermatomed or heat-separated epidermis, depending on the objective. The permeation profile is typically expressed as the cumulative amount of drug permeated per unit area versus time, from which key parameters such as steady-state flux (Jss) and permeability coefficient (Kp) describing drug transport across the skin barrier are derived.

Jss represents the rate at which the drug permeates through the membrane at equilibrium and is calculated using the following equation:

$$J_{ss}=(dQ/dt)/A$$

where dQ/dt is the rate of drug permeation and A is the effective diffusion area. Flux is an important parameter for assessing the efficiency of drug transport across the skin, particularly for vesicular carriers designed to enhance penetration.

Kp describes the intrinsic permeability of the membrane to the drug and is determined using the following relationship:

$$K_{p}=J_{ss}/C_{0}$$

where C_0_ is the initial drug concentration in the donor compartment. This parameter reflects the ability of the formulation to facilitate drug diffusion through the skin barrier.

Another important parameter is the lag time (t_L_), which corresponds to the time required for the drug to establish steady-state permeation and is obtained from the x-intercept of the linear region of the permeation curve. Lag time is commonly approximated using the diffusion model:

$$t_{L}\approx h^{2}/(6D),$$

where h is the membrane thickness and D is the diffusion coefficient of the drug within the membrane [233]. These parameters collectively provide quantitative insight into the delivery and absorption characteristics of ultradeformable vesicles, enabling comparison of formulations and evaluation of their ability to enhance drug transport through the stratum corneum. In wound healing applications, improved flux and permeability combined with reduced lag time indicate more efficient drug deposition at the wound site, which can contribute to enhanced therapeutic outcomes in topical treatment. Since wound conditions alter barrier properties, many studies use injured or disease relevant membranes, including standardized excision wounded skin, thermally injured or burned skin, or infected wound tissue, for example, tissue inoculated with *Staphylococcus aureus* or MRSA [234]. Diabetic relevance is modeled using skin from diabetic animals and or diabetic excision wounds. Where biological tissue is limited or high reproducibility is required, artificial or reconstructed human skin equivalents, such as reconstructed epidermis or full-thickness models, are used as supportive screening tools [235]. Local targeting is confirmed by skin retention and deposition studies such as tape stripping, where sequential adhesive tapes remove the SC layer by layer, followed by extraction of the drug from tape strips and from the remaining viable skin for quantification. This approach helps differentiate superficial surface loading from true intradermal delivery, an important distinction in diabetic wounds where healing is impaired. In vivo microdialysis can measure the amount of unbound drug in dermal interstitial fluid, and the vasoconstrictor assay provides a pharmacodynamic indicator of cutaneous delivery via the blanching response [236]. Depthwise distribution is further visualized by confocal laser scanning microscopy using fluorescent probes or ruthenium-based dyes to map penetration pathways and localization within superficial versus viable layers relevant to wound repair. Ruthenium-based dyes are used in some protocols as contrast and penetration indicators [237]. Local skin retention or dermatopharmacokinetics is also assessed through time-dependent skin sampling to calculate the C_max-skin_, T_max_, AUC_skin_ and retention time [238]. These parameters indicate whether nanovesicles can sustain therapeutically effective drug levels in the skin for a longer time. The prolonged local exposure is especially valuable in diabetic wound management, where healing is delayed and the wound microenvironment can hinder repair, making sustained drug presence important. Mechanistic confirmation of barrier interaction/lipid fluidization can be obtained by attenuated total reflection–Fourier transform infrared spectroscopy, which tracks changes such as CH_2_ stretching peak shifts and lipid order indices, supporting enhanced permeation driven by ethanol and by edge activators [239]. Recent advances in imaging technologies have enabled improved characterization of wound healing processes and evaluation of topical drug delivery systems. Optical coherence tomography allows noninvasive visualization of skin structure, re-epithelialization, and granulation tissue formation, while the high-resolution imaging technique angiography enables assessment of microvascular development during healing [240,241]. Photoacoustic imaging provides functional information on tissue oxygenation, hemoglobin levels, and vascular perfusion, supporting the evaluation of angiogenesis and tissue regeneration [242,243]. At the microscopic level, multiphoton microscopy, including two-photon fluorescence and second-harmonic generation, facilitates label-free imaging of collagen organization and extracellular matrix remodeling during wound healing [244,245]. Fluorescence lifetime imaging microscopy further enables the analysis of cellular metabolic activity by detecting changes in endogenous fluorophores associated with tissue repair processes [246]. In addition, Raman spectroscopy and coherent anti-Stokes Raman scattering microscopy provide biochemical insights into tissue composition and nanocarrier–skin interactions, including lipid-based vesicle behavior [247,248]. Hyperspectral imaging further supports noninvasive assessment of tissue oxygenation, inflammation, and infection in wounds [249]. Recently, microscopic hyperspectral infrared spectroscopy imaging has been used to obtain detailed chemical and heterogeneity information from the SC, thereby overcoming the limitations of macroscopic analysis using spectroscopy [250]. Because wound therapy demands high tolerability, the evaluation is commonly extended to skin irritation and cytotoxicity. Dermal irritation may be screened by Draize scoring of erythema and edema [251]; the Hen’s Egg Test on chorioallantoic membrane vascular reactions such as haemorrhage, lysis and coagulation time; or reconstructed human epidermis endpoints such as tissue viability and IL-1α release [252]. In parallel, human keratinocyte assays using 3-(4,5-dimethylthiazol-2-yl)-2,5-diphenyltetrazolium bromide, sulforhodamine B, or Alamar Blue quantify percent viability, IC_50_ and morphological changes for early dermal safety screening, ensuring that enhanced penetration, especially with transethosomes or transferosomes, does not come at the cost of unacceptable irritation or cytotoxicity [188].

### 8.2. In Vivo

Ultradeformable vesicles are evaluated in wound models using endpoints for healing rate, tissue regeneration and mechanism of action (Table 5). Excision and diabetic wounds emphasize wound closure (planimetry) and histology-confirmed epithelialization, supported by collagen estimation (hydroxyproline), granulation tissue quality, and tensile strength, along with confocal microscopy-based penetration depth and spatial distribution. Burn wound assessment focuses on re-epithelialization, collagen remodeling/scar architecture and angiogenesis, in addition to vesicle deformability testing to support the squeeze-through mechanism. In infected wound models, evaluation prioritizes microbial burden (CFU counts) and infection monitoring, with optional imaging to assess colonization/biofilm-like structures.

## 9. Critical Perspectives

Ultradeformable nanovesicles, ethosomes, transethosomes and transferosomes have strong future potential in wound care. This is because their enhanced deformability/penetration can increase local deposition of antimicrobials, anti-inflammatory agents, antioxidants and pro-regenerative payloads within complex wound beds, supporting faster closure and better tissue repair across different types of wounds [188]. Looking forward, key prospects include multifunctional systems with early anti-infective/antibiofilm action followed by pro-angiogenic and matrix remodeling support. Hybrid designs where vesicles are incorporated into gels/hydrogels, films, or advanced dressings can prolong residence time and enable sustained release, an approach particularly relevant for chronic and high-exudate wounds such as diabetic ulcers and burns [253]. For diabetic wounds, vesicle-enabled delivery is promising because diabetic healing is impaired by chronic inflammation, oxidative stress and inadequate angiogenesis, mechanisms that often require combination therapy and sustained local exposure rather than short-contact topical dosing. Furthermore, evaluation in models that closely mimic real clinical conditions, such as biofilm-associated and diabetic ischemic wounds, is essential. Such clinically relevant study designs will facilitate the effective translation of these systems into real-world therapeutic applications. Rapid advances have been made in sensor-integrated and stimulus-responsive wound dressings capable of monitoring parameters such as pH, temperature, moisture and infection biomarkers, while enabling on-demand therapeutic release and supporting artificial intelligence-assisted wound assessment [107].

## 10. Translational Challenges

Clinical translation faces several practical challenges, including colloidal and chemical instability, drug leakage, difficulties in scale-up, and ensuring batch-to-batch reproducibility. Additional limitations include sterilization constraints that may disrupt vesicle structure and performance variability in real wound environments influenced by pH shifts, enzymes, heavy exudate, and bacterial burden [200]. In addition, robust clinical relevance still depends on better standardization of characterization and stronger efficacy evidence in models that mimic biofilm-dominant infected wounds and ischemic diabetic wounds, alongside safety assessment for irritation/sensitization and systemic exposure when used on large-area burns. Only one relevant patent application has been identified so far (application no. 202211051495, published 9 September 2022) describing a method to formulate and evaluate atorvastatin ethosomal gel for diabetic wounds. At present, no clinical trials have been reported for the ultradeformable vesicle gel-based approach; therefore, its safety, optimal dosing, clinically relevant endpoints and comparative effectiveness remain unproven in humans. Large-scale manufacturing and reproducibility are significant concerns, as commonly used laboratory preparation methods (e.g., thin-film hydration) may lead to variability in vesicle size, encapsulation efficiency, and batch-to-batch consistency when scaled up. Formulation stability is another critical issue, since the presence of high ethanol or surfactant concentrations may cause vesicle aggregation, drug leakage, or physicochemical instability during storage. Strategies such as lyophilization and pro-vesicular systems have been explored to enhance stability and shelf life. In addition, regulatory challenges persist because standardized regulatory frameworks for nanocarrier-based topical systems are still evolving, particularly regarding physicochemical characterization, quality control, and long-term safety evaluation [254]. The translational gap is primarily driven by stringent regulatory and Chemistry, Manufacturing and Controls requirements, including the need for GMP-scalable manufacturing, tight control of critical quality attributes, long-term stability, suitable packaging and compliance with microbial and preservative standards [255]. The use of ethanol and edge activators complicates development owing to concerns about skin irritation, barrier disruption and systemic absorption, especially in damaged skin. To bridge this gap, thorough safety evaluations and well-designed clinical trials need to be conducted to promote the clinical approval of ultradeformable vesicular dermatological formulations. Additional challenges include regulatory complexity for combination products (e.g., vesicle-in-dressing platforms) and the need to ensure reproducible characterization of critical parameters such as vesicle size, elasticity, entrapment efficiency, and in vitro–in vivo correlation. Addressing these challenges will be essential to facilitate the successful translation of ultradeformable vesicular systems into clinically viable wound healing therapies.

## 11. Conclusions

This review highlights ultradeformable vesicles as promising delivery systems for infected, burn, diabetic and excision wounds by enabling phase-specific delivery of therapeutics across the wound healing cascade. It summarizes the composition and key formulation variables of major elastic vesicles and links these properties to wound healing applications, in vitro characterization and in vivo outcomes. Overall, many studies report improved wound closure, reduced microbial burden and enhanced tissue repair, especially when vesicles are incorporated into gels or advanced dressings for better retention and sustained release. However, clinical translation is still limited by unstandardized testing and reporting, variability in models and endpoints, and practical hurdles such as stability/drug leakage, sterilization constraints and scale-up reproducibility.

## Figures and Tables

**Table 1:** Comparison of ethosomes, transferosomes and transethosomes.

Property	Ethosomes	Transferosomes	Transethosomes
Description	Soft phospholipid nanovesicles with a relatively high ethanol content (~20–45% *v*/*v*) for fluidization and water, often combined with propylene glycol. Designed to enhance dermal/transdermal drug delivery by increasing both skin permeability and vesicle flexibility.	Ultradeformable (elastic) lipid vesicles composed of phospholipids and an edge activator for high flexibility. Deformability enables passage through narrow skin pores without breaking, aided by the transdermal hydration/osmotic gradient.	Advanced ethosomal vesicles composed of phospholipids, ethanol, and an edge activator (surfactant) and/or other permeation enhancers. This unique combination imparts higher vesicle deformability and significantly improves skin permeation, enabling deeper drug delivery into the skin layers compared with conventional ethosomes or transferosomes.
Differentiation	Characterized by a high ethanol content, which plays a critical role in enhancing skin permeation. Ethanol disrupts the lipid organization of the stratum corneum and increases the ethosomes’ fluidity, leading to increased permeability, allowing them to permeate the skin more effectively than conventional liposomes. Relatively soft and flexible, they are generally less elastic than transferosomes due to the absence of edge activators in their formulation.	Contains an edge activator as the key component, without ethanol. Permeates the skin primarily through extreme membrane elasticity combined with hydration- or osmotic-driven transport via skin microchannels. Ethanol is absent or present only in low amounts, with skin permeation relying mainly on vesicle deformability by surfactant rather than lipid disruption.	Vesicular systems incorporating ethanol and an edge activator within the same vesicular system. Demonstrate higher drug entrapment efficiency. Dual composition produces synergistic enhancement of vesicle deformability and skin permeation. Often achieve higher skin deposition and transdermal drug flux than either conventional ethosomes or transferosomes for a wide range of active compounds.
Advantages	High skin permeation and drug deposition for many hydrophilic and lipophilic drugs. High drug loading/solubilization due to ethanol. Relatively simple, scalable preparation.	Exceptional deformability allows delivery through intact stratum corneum. Can deliver wide range of actives (including peptides/proteins) with reduced invasiveness. Often enable high skin permeation at low drug dose; suitable for controlled/targeted delivery.	Greater deformability and permeation than ethosomes due to edge activator and ethanol. Often improve delivery of larger/less permeable molecules. Can enhance both skin retention (topical) and transdermal flux depending on composition.
Disadvantages	High ethanol may cause irritation/dryness in sensitive skin. Volatility can affect composition if not well sealed. Some formulations show drug leakage or vesicle fusion on storage.	Edge activators may irritate skin and can destabilize membranes if used at high levels. Often less physically stable (aggregation/leakage) and sensitive to processing conditions. Need careful optimization and storage. Difficult to load hydrophobic drugs.	More complex composition (ethanol and surfactant) may increase irritation potential and require careful safety evaluation. Surfactant/ethanol can increase drug leakage. Stability depends strongly on formulation variables. High cost.
Method of preparation	Cold method (most common)/hot method.	Thin-film hydration (most common), reverse-phase evaporation and ethanol injection (less common).	Cold method (most common)/hot method.

**Table 2:** Components used in the formulation of ethosomal, transferosomal, and transethosomal systems.

Component Category	Vesicular System	Examples	Role in Vesicles
Phospholipids	Ethosomes, transferosomes, transethosomes	Soybean-derived phosphatidylcholine (PC) (Phospholipon^®^ 90G, Phospholipon^®^ 80H, Lipoid^®^ S 100, Lipoid^®^ S 75), semi-synthetic PC (dipalmitoyl PC, distearoyl PC), egg-derived PC (Lipoid^®^ E 80, Lipoid^®^ E 75), hydrogenated phospholipids (Phospholipon^®^ 90H, Phospholipon^®^ 80H), lecithin (soy lecithin Epikuron^®^ 200/Epikuron^®^ 145V)	Form vesicular bilayer, enhance drug entrapment and interaction with stratum corneum (SC) lipids, improve vesicle stability and rigidity.
Alcohol	Ethosomes, transethosomes	Ethanol, isopropyl alcohol, propylene glycol	Improves membrane fluidity and deformability, solubilizes drugs and enhances skin permeation.
Edge activators (surfactants)	Transferosomes, transethosomes	Nonionic (tween 80, tween 20, span 60, Cremophor EL), ionic bile salts (sodium cholate, sodium deoxycholate), cationic (cetyltrimethylammonium bromide, dimethyldidodecylammonium bromide)	Impart ultradeformability and elasticity to vesicles, enhancing transdermal permeation.
Aqueous phase	Ethosomes, transferosomes, transethosomes	Purified water, phosphate buffer	Hydration medium for vesicle formation, influences pH and stability.
Permeation enhancers	Ethosomes, transethosomes	Fatty acids (oleic acid), terpenes (menthol, limonene, iso-eucalyptol), glycols (propylene glycol), polar aprotic organic solvent (dimethyl sulfoxide) and dendritic polymer (polyamidoamine G3), volatile oil (eucalyptus oil, peppermint oil, turpentine oil, alcohol (ethanol), anionic surfactant (sodium lauryl sulphate), glycol ether (transcutol)	Fluidize vesicular and skin lipids and disrupt SC lipid structure, thereby facilitating enhanced skin drug permeation.
Membrane stabilizers (optional)	Ethosomes, transferosomes, transethosomes	Cholesterol	Enhance vesicle stability and reduce drug leakage. Excessive amounts may reduce deformability.
Charge-inducing agents (optional)	Ethosomes, transferosomes, transethosomes	Dicetyl phosphate, cetyltrimethylammonium bromide	Modify surface charge to improve vesicle stability and skin interaction.
Cryoprotectants (optional)	Ethosomes, transferosomes, Transethosomes	Trehalose, mannitol, sucrose	Protect vesicles during freeze-drying and prevent aggregation and fusion during storage.
Gelling agents (optional)	Ethosomes, transferosomes, transethosomes	Carbopol 934/940, hydroxypropyl methylcellulose (HPMC)	Increase viscosity and skin retention and improve patient compliance and controlled release.
Neutralizers/pH adjusters (optional)	Ethosomes, transferosomes, transethosomes	Triethanolamine, sodium hydroxide	Neutralize carbopol and adjust pH for skin compatibility.

**Table 3:** Mini-database summarizing formulation characteristics and therapeutic outcomes of ethosomal formulations used in wound healing.

Disease/Disorder	Drug/Phytochemical	Composition	Particle Size, Zeta Potential, Polydispersity Index, Encapsulation Efficiency	Key Functional Outcome	Wound Healing Outcome	Reference
Excision wound healing	Curcuma longa	Soya phosphatidyl choline, ethanol, tween 80, sodium lauryl sulphate	34.8 to 371 nm, 23.2 ± 7.47–36.8 ± 7.47 mV, 0.5 ± 0.35–1.2 ± 0.35, 67.76 ± 7.34%	Methanolic extract exhibited greater antibacterial activity than the ethanolic and aqueous extracts.	Improved wound contraction at a dose rate of 0.5 g/cm^2^ and 1.0 g/cm^2^, reduced pain and enhanced granulation tissue formation compared with crude extract.	[156]
Microbial wound healing	Garlic essential oil	Soya PC, sodium lauryl sulphate and tween 80	91.28–871.10 nm, 0.273, −19.6 mV, 98.58%	Strong antibacterial and antifungal activity in guinea pig and mouse models.	Enhanced epithelial regeneration, collagen deposition, fibroblast proliferation and neovascularization with 0. 5% garlic oil comparable to standard, povidone–iodine.	[157]
Diabetic wound healing	Kaempferol	Soya lecithin, ethanol, propylene glycol, carbopol 934, cholesterol	186.8 nm, 31.9 mV, 0.285, 96.2%	Enhanced antimicrobial activity against MRSA and improved drug delivery (≈2-fold pharmacokinetic increase).	Improved re-epithelialization, reduced ulceration and accelerated wound closure in Wistar albino diabetic rat models.	[158]
Burn wound healing	Curcumin	Egg lecithin, cholesterol, ethanol, carbopol	85%	Enhanced antibacterial activity and improved formulation stability compared to free curcumin. Demonstrated efficacy against burn wound bacterial flora comparable to silver sulfadiazine cream.	Accelerated re-epithelialization (*p* < 0.01), neovascularization (*p* < 0.01), collagen synthesis *p* < 0.001), and granulation tissue formation (*p* < 0.001), with complete wound contraction by day 16.	[147]
Burn wound healing	Thymosin β-4	l-alpha-PC from soybean, cholesterol, ethanol, sodium deoxycholate, carbomer 934	127.8 ± 3.2 nm, −25.1 ± 2.8 mV, 63.2 ± 4.5%	1.67-fold higher cumulative drug release in vitro than that of the T-β4 gel.	Reduced wound healing time by half compared with conventional gel in second-degree burn model.	[159]

**Table 4:** Mini-database summarizing the composition, functional characteristics, and wound healing outcomes of transferosome-based gel formulations.

Disease/Disorder	Drug/Phytochemical	Composition	Particle Size, Zeta Potential, Polydispersity Index, Encapsulation Efficiency	Key Functional Outcome	Wound Healing Outcome	Reference
Excision wound healing	Centella asiatica	Cholesterol, sorbitan oleate, soyabean lecithin, propylene glycol, tween 80, tocopherol acetate, ammonium acryloyl dimethyltaurate/VP copolymer (aristoflex avc)	135.22 ± 4.80 nm, −26.13 ± 0.58 mV, 0.22 ± 0.01, 68% (madecassoside) and 89% (asiaticoside)	Enhanced skin permeation vs. liposomes and niosomes due to high deformability index (1.31 ± 0.21 mg/cm^2^).	Increased fibroblast proliferation (91.9%) and collagen synthesis (213.3%). Reduced inflammation and improved epithelial regeneration over 21 days, with wound closure rates comparable to fibroblast growth factor.	[195]
Excision wound healing	Sesamol	Tween 80, span 80, carbopol 940	272 ± 1.04 nm, −28.65 mV, 70 ± 1.90%	Improved skin penetration and deposition. Highest deformability index value (28.6 ± 1.08) was obtained with Tween-80 vesicles.	Enhanced wound contraction and improved histological architecture after 21 days of treatment.	[196]
Excision wound healing	Calendula officinalis	PC, cholesterol, span 80, dimethyl sulfoxide, propylene glycol, wool fat, hard paraffin, cetostearyl alcohol, soft paraffin	110.5 ± 5.2 nm, −32.5 ± 2.8 mV, 0.214 ± 0.03, 82.4 ± 2.1%	Sustained drug release (88.2% over 24 h) following zero-order kinetics.	Enhanced wound contraction (98.7% by day 14), supported by histopathological evidence of organized collagen deposition, reduced inflammation and complete re-epithelialization.	[197]
Microbial wound healing	Rhodomyrtus tomentosa	Lecithin, cholesterol	405.3 ± 2.0 nm, −61.62 ± 0.86 mV, 0.16 ± 0.08, 81.90 ± 0.31%	Stable nanosized vesicles with antioxidant potential. Free radical scavenging assay showed high scavenging activity against DPPH and ABTS radicals.	Broad-spectrum antimicrobial activity with minimum inhibitory and minimum bactericidal concentrations at 8–256 and 64–1024 μg/mL, respectively. Significant NO inhibition.	[198]
Diabetic wound healing	Azadirachta indica, Ocimum sanctum and Allium sativum	Phospholipid, span 60, tween 80, carbopol 940	165 ± 2.5 nm, −35.0 ± 1.2 mV, 0.20, 85.6 ± 1.7%	Sustained drug release over 24 h, good physical and chemical stability. Good extrudability (4.2–5.1 g/cm^2^), indicating superior dispensing characteristics.	92.3% wound contraction in diabetic rats by day 21.	[199]
Burn wound healing	Fusidic acid	PC S100, span 80, cholesterol, carbopol 934	145.16 nm, −42.7 mV, 0.269, 87.47 ± 0.119%	Sustained drug release following Fickian diffusion, with 47% and 94% drug release at 6 h and 24 h respectively.	Potential antibacterial wound healing.	[184]

**Table 5:** Summary of wound healing evaluation parameters used for assessing ultradeformable vesicle-based formulations in different disease models.

Wound Type/Model (Examples)	Evaluation Property	Method/Instrument	Principle	Evaluation Parameter	Significance
Excision wound (full thickness circular/elliptical)	Wound closure/contraction	Digital photography + digital planimetry	Calibrated images used to compute wound area over time	Wound area (cm^2^), % closure, % contraction, healing rate	Primary efficacy endpoint for topical formulations in excisional wounds
	Epithelialization	Visual scoring + histology (H&E)	Endpoint = complete epithelial cover, histology shows epithelial migration	Epithelialization period (days), epithelial gap, epidermal thickness	Indicates barrier restoration, complements contraction
	Mechanical strength	Tensile strength testing (tensiometer/UTM)	Measures breaking force of healed tissue (commonly incision model)	Breaking strength (N), tensile strength (MPa)	Functional outcome: stronger healed tissue = better remodeling
	Collagen deposition (biochemical)	Hydroxyproline assay	Hydroxyproline quantifies collagen content	Hydroxyproline (µg/mg tissue), total collagen estimates	Objective measure supporting histology, linked to tensile strength
	Granulation tissue quality	Histology (H&E) + morphometry	Measures granulation thickness/cellularity and tissue organization	Granulation thickness, fibroblast density and inflammatory score	Shows proliferative phase quality and wound bed maturity
	Skin penetration depth and spatial distribution	Confocal laser scanning microscopy using fluorescent probe-labeled vesicles/drug	Optical sectioning visualizes depthwise penetration of skin layers	Penetration depth (µm), fluorescence intensity vs. depth, distribution in epidermis/dermis	Direct evidence that ultra deformable vesicles can deliver payloads beyond SC, often used to compare vesicle systems
Burn wound (partial/full thickness thermal burn)	Re-epithelialization	Histology (H&E) ± immunostaining	Tissue sections show epithelial regeneration over burn	% re-epithelialization, epidermal thickness	Core outcome in burn healing indicates restoration of skin surface
	Collagen remodeling/scar architecture	Collagen imaging/histology (e.g., collagen fiber assessment)	Collagen organization and density assessed via imaging/staining	Collagen signal/intensity, fiber alignment, scar features	Scar quality and remodeling are critical in burns
	Angiogenesis/vascularization	Vessel imaging/quantification (e.g., vascular density)	Quantifies neovascularization in the burn area	Vessel density, angiogenesis markers	Angiogenesis supports granulation and repair
	Vesicle deformability	Extrusion method (forced through membrane) + size measurement	Ultradeformable vesicles pass through pores smaller than their diameter	Deformability index/elasticity, size before/after extrusion	Supports the squeeze-through mechanism, key differentiator for transethosomes (edge activator effect)
Infected wound (infected excision/burn, mono- or polymicrobial)	Microbial burden	Total Viable Count (TVC)/Colony Forming Units (CFU) swab/tissue homogenate culture; CFU counting	Quantifies viable bacteria	CFU/g tissue, log reduction, species counts	Key endpoint distinguishing healing from antimicrobial efficacy
	Infection status and monitoring	Clinical infection frameworks/qualitative–quantitative monitoring	Infection progresses along contamination, colonization, infection continuum	Local vs. spreading infection signs, monitoring response	Ensures correct interpretation and antimicrobial stewardship
	Biofilm/tissue invasion (optional, mechanistic)	Microscopy/SEM or model-specific imaging	Visualizes bacterial colonization/biofilm-like structures	Colonization patterns, biofilm indicators	Explains delayed healing and treatment failure in chronic infection
Diabetic wound (STZ-induced, Db/db mice, high-fat/STZ combos)	Delayed closure (impaired healing)	Digital planimetry + time to closure	Same as excision closure but in impaired-healing context	% closure, delayed healing rate vs. control	Demonstrates performance under clinically relevant impairment
	Inflammation and cytokine dysregulation	ELISA/RT-qPCR/Western blot	Quantifies inflammatory mediators/growth factors	IL-6, TNF-α, IL-10, growth factors, signaling proteins	Mechanistic proof: diabetic wounds are inflammation-prone and slow to resolve
	Oxidative stress/antioxidant status	MDA, SOD, CAT, GPx assays	Measures oxidative damage and antioxidant defenses	MDA, SOD/CAT/GPx activity	Supports mechanisms (oxidative stress contributes to impaired healing)
	Histopathology (granulation, collagen, epithelialization)	Histology + scoring	Tissue architecture shows delayed granulation and remodeling	Granulation thickness, collagen deposition, epithelial gap	Complements closure data, shows quality of repair

## Data Availability

No new data were created or analyzed in this study.
